# Bioenergetic Inhibitors: Antibiotic Efficacy and Mechanisms of Action in *Mycobacterium tuberculosis*

**DOI:** 10.3389/fcimb.2020.611683

**Published:** 2021-01-11

**Authors:** Erik J. Hasenoehrl, Thomas J. Wiggins, Michael Berney

**Affiliations:** Department of Microbiology and Immunology, Albert Einstein College of Medicine, Bronx, NY, United States

**Keywords:** *Mycobacterium tuberculosis*, bioenergetics, bactericidal, electron transport chain, bedaquiline, Q203, persistence

## Abstract

Development of novel anti-tuberculosis combination regimens that increase efficacy and reduce treatment timelines will improve patient compliance, limit side-effects, reduce costs, and enhance cure rates. Such advancements would significantly improve the global TB burden and reduce drug resistance acquisition. Bioenergetics has received considerable attention in recent years as a fertile area for anti-tuberculosis drug discovery. Targeting the electron transport chain (ETC) and oxidative phosphorylation machinery promises not only to kill growing cells but also metabolically dormant bacilli that are inherently more drug tolerant. Over the last two decades, a broad array of drugs targeting various ETC components have been developed. Here, we provide a focused review of the current state of art of bioenergetic inhibitors of *Mtb* with an in-depth analysis of the metabolic and bioenergetic disruptions caused by specific target inhibition as well as their synergistic and antagonistic interactions with other drugs. This foundation is then used to explore the reigning theories on the mechanisms of antibiotic-induced cell death and we discuss how bioenergetic inhibitors in particular fail to be adequately described by these models. These discussions lead us to develop a clear roadmap for new lines of investigation to better understand the mechanisms of action of these drugs with complex mechanisms as well as how to leverage that knowledge for the development of novel, rationally-designed combination therapies to cure TB.

## Introduction

The World Health Organization’s (WHO) End TB strategy was developed in 2015 to end the tuberculosis (TB) epidemic by 2035 (WHO, 2019). This strategy has set the goal of reducing global TB incidence and disease burden rates to levels consistent with developed nations with universal health care. Unfortunately, TB incidence was reduced by only 6.3% between 2015 and 2018, which is well short of the total reduction of 20% required by 2020 to meet projected targets (WHO, 2019). Further compounding the difficulties in eradication is the growing incidence of multi-drug resistant (MDR) TB. In 2018 there were approximately 500,000 new cases of rifampicin resistant TB, of which 78% were MDR (WHO, 2019). Even more alarming is that only 56% of those treated for MDR-TB were actually cured (WHO, 2019). This latter statistic underscores the dire need for therapeutic regimens capable of treating MDR-TB and preventing drug-resistance acquisition (WHO, 2019).

One of the major contributors to both the continued disease burden and the development of drug resistance is the prolonged treatment timelines required to cure tuberculosis infection. Drug-susceptible (DS) infections traditionally required six months of treatment with first line chemotherapeutics (WHO, 2019). Excitingly, recent phase 3 clinical trials found that specific combination therapies were effective at reducing required treatment timelines for DS-TB to 4 months, and for MDR-TB to 6 months (Conradie et al., 2020a; WHO, 2020). However, resistance has already been observed for many of the drugs included in these regimens and these timelines are still much longer than treatment for most other bacterial infections (WHO, 2019). Such lengthy regimens can lead to non-compliance for a variety of reasons, which ultimately leads to treatment failure and fosters further drug resistance acquisition (Pablos-Méndez et al., 1997; Dorman and Chaisson, 2007). This protracted treatment course is likely required because of multiple obstacles to therapeutic efficacy, including drug tolerance of the bacterium and poor pharmacodynamics at the site of infection (Gomez and McKinney, 2004; Barry et al., 2009; Dartois, 2014). Drug tolerance, the focus of this review, is proposed to be due to the ability of *Mtb* to enter a metabolically quiescent state known as persistence where it is phenotypically tolerant to drug challenge with conventional chemotherapeutics (Gomez and McKinney, 2004; Dhar and McKinney, 2007; Dorman and Chaisson, 2007). These conventional drugs, including isoniazid, rifampicin, ethambutol, fluoroquinolones and aminoglycosides, among others, target biomass generating processes like nucleic acid, protein and cell wall biosynthesis. When mycobacteria assume a dormant metabolic state, they become more tolerant to these drugs as they are non-reliant on the generation of these biomolecules (Wayne and Hayes, 1996; Betts et al., 2002; Rao et al., 2008; Gengenbacher et al., 2010; Keren et al., 2011). Drug efficacy then requires extensive therapy in order to slowly kill these cells as they stochastically reactivate their metabolisms and become susceptible to drugs once again (Zhang et al., 2012b). However, more recent efforts in drug development have identified alternative metabolic pathways, which remain essential for *Mycobacterium tuberculosis (Mtb)* even during metabolic dormancy, thus promising hope for the development of new drug regimens capable of quickly curing TB infections.

**Figure 1:**
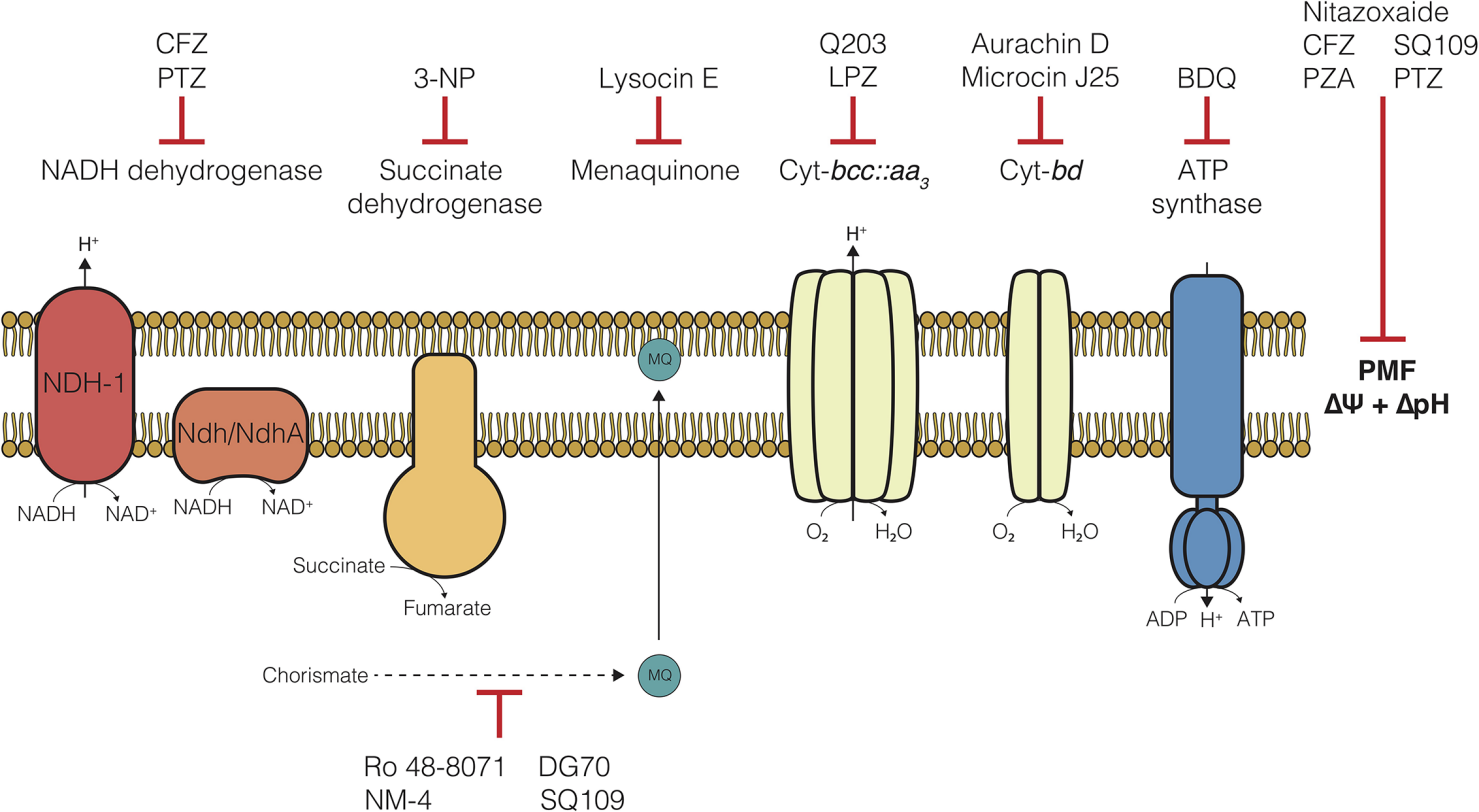
The electron transport chain of *Mycobacterium tuberculosis* and compounds targeting each component. Clofazimine (CFZ), Phenothiazines (PTZ), 3-nitropropionate (3-NP), Lansoprazole (LPZ), Bedaquiline (BDQ), Pyrazinamide (PZA).

In 2012, the FDA approved Bedaquiline (BDQ), an inhibitor of the F_1_F_O_-ATP synthase, for the treatment of TB. Of note, was the apparent ability of BDQ to kill metabolically quiescent cells, where traditional chemotherapeutics targeting biosynthetic processes could not (Koul et al., 2008; Rao et al., 2008; Gengenbacher et al., 2010). This effectively launched a new age of antimycobacterial drug development and led to a deluge of studies focused on development of inhibitors of enzymes in the electron transport chain (ETC) (Bald et al., 2017; Cook et al., 2017; Hards and Cook, 2018; Wellington and Hung, 2018; Appetecchia et al., 2020; Foo et al., 2020). The ETC is a collection of membrane-bound and -associated enzymes and cofactors that are the primary cellular drivers responsible for recycling reduced substrates from central carbon metabolism, generating a proton motive force (PMF) essential for maintaining transmembrane electrochemical gradients, and ATP synthesis *via* oxidative phosphorylation (**Figure 1**). Indeed, each of these processes have been found to be required during persistence in *Mtb* (Rao et al., 2008; Gengenbacher et al., 2010). Specifically, these mechanisms are essential in metabolically dormant cells induced by environmental stressors, such as hypoxia or nutrient starvation (Rao et al., 2008; Gengenbacher et al., 2010), and referred to in this review as non-replicating persister (NRP) cells. Over the last two decades, a broad array of drugs targeting various ETC components have been found to kill these drug tolerant NRP cells through disruption of these essential processes. Some bioenergetic inhibitors are also effective against drug-induced persister cell populations, which are a subset of a larger culture that survives drug challenge (Grant et al., 2012; Cohen et al., 2013). However, this specific population and the drugs which effectively kill these cells are not the focus of this review.

Here, we provide a focused review of the current state of art of bioenergetic inhibitors of *Mtb* with an in-depth analysis of the metabolic and bioenergetic disruptions caused by specific target inhibition. We also catalogue efforts to date to evaluate synergistic and antagonistic drug interactions with bioenergetic inhibitors. We then use this foundation to explore the reigning theories on the mechanisms of antibiotic-induced cell death and discuss how bioenergetic inhibitors in particular fail to be adequately described by these models. These discussions lead us to develop a clear roadmap for new lines of investigation to better understand the mechanisms of action of these drugs with complex mechanisms as well as how to leverage that knowledge for the development of novel, rationally designed combination therapies to cure TB.

## Current State of Bioenergetic Inhibitors

Since the advent of bedaquiline in 2012, the collection of small molecule inhibitors of bioenergetics has expanded dramatically. The inhibitors identified in these studies have now become a major component (>30%) of all new antimycobacterial drugs in clinical trials and are included in more than 65% of Phase III trial regimens (**Table 1**) (WHO, 2019). Along with many pre-clinical candidates, these drugs mostly target a variety of enzymes involved in oxidative phosphorylation, including the F_1_F_O_-ATP-synthase, cytochrome oxidases, NADH dehydrogenases, succinate dehydrogenases, and menaquinone biosynthesis (**Figure 1**). Some of these drugs also display broad mechanisms of action which target multiple processes, both within the ETC and without. A foundational and complete accounting of oxidative phosphorylation enzymes and their physiology can be found by (Cook et al., 2014), and a comprehensive review of drug classes targeting the ETC can be found by (Foo et al., 2020).

**Table 1:** Table of new drugs currently in clinical trials to treat *Mycobacterium tuberculosis* (WHO, 2019).

Drug	Compound	Target	Class	Clinical Status	New/Old
BDQ	Diarylquinolone	ATP-Synthase inhibitor / Uncoupler	ETC	Phase III	New
BTZ-043	Benzothizinone	DprE1 inhibitor	Cell wall	Phase I	New
Contezolid (MRX4/1)	Oxazolidinone (linezolid)	23s rRNA	Protein Synthesis	Phase I	New
Delaminid	Nitroimidazole	Mycolic Acid inhibition / NO Toxicity	ETC/Cell Wall	Phase III	New
Delpazolid	Oxazolidinone	23s rRNA	Protein Synthesis	Phase II	New
GSK-3036656	Oxaborole	Luecyl-tRNA Synthetase	Protein Synthesis	Phase I	New
Macozinone (PBTZ169)	benzothizinone	DprE1 inhibitor	Cell Wall	Phase I	New
OPC-167832	Cabostyril derivative	DprE1 inhibitor	Cell Wall	Phase I	New
Pretomanid	Nitroimidazole	Mycolic Acid inhibition / NO Toxicity	ETC/Cell Wall	Phase III	New
Telecebec (Q203)	Imidazopyridine	Cyt-bc1:aa3	ETC	Phase II	New
SPR720	Benzimidazolle	DNA Gyrase B (GyrB)	DNA Synthesis	Phase I	New
SQ109	Ethylenediamine	Menaquinone synthesis/MmpL3	ETC	Phase IIb/III	New
Sutezolid (PNU-100480)	Oxazolidinone (linezolid)	23s rRNA	Protein Synthesis	Phase IIa	New
TBA-7371	Azaindole	DprE1 inhibitor	Cell wall	Phase I	New
TBI-166	Riminophenazine (Clofazimine)	ROS Produciton (Via NADH)	ETC	Phase I	New
TBI-223	Oxazolidinone	23s rRNA	Protein Synthesis	Phase I	New
Clofazimine	Riminophenazine	ROS Produciton (Via NADH)	ETC	Phase III	Old
Levofloxacin	Fluoroquinolone	Topoisomerase II/IV, DNA Gyrase	DNA Synthesis	Phase II	Old
Linezolid	Oxazolidinone	23s rRNA	Protein Synthesis	Phase III	Old
Moxifolxacin	Fluoroquinolone	Topoisomerase II/IV, DNA Gyrase	DNA Synthesis	Phase III	Old
Nitazoxanide	Nitrothiazolyl-salicylamide	Membrane Potential / pH Homeostasis	ETC	Phase II	Old
Rifampicin (High dose)	Rifamycin	RpoB RNA Polymerase	DNA Synthesis	Phase III	Old
Rifapentine	Rifamycin	RpoB RNA Polymerase	DNA Synthesis	Phase III	Old

### ATP Synthase Inhibitors

The F_1_F_O_-ATP synthase (encoded by *atpBEFHAGDC*) catalyzes the final step of oxidative phosphorylation. It is responsible for the synthesis of ATP from ADP and inorganic phosphate by leveraging the potential energy from the proton gradient generated by respiration in the ETC (Cook et al., 2014). The F_1_F_O_-ATP synthase has been found to be essential for growth, as well as survival during hypoxic and starvation-induced NRP (Tran and Cook, 2005; Rao et al., 2008; Gengenbacher et al., 2010). BDQ (also known as R207910, TMC207, and Sirturo) is the prototypical inhibitor of oxidative phosphorylation *via* its disruption of the mycobacterial F_1_F_O_-ATP synthase (Andries et al., 2005). It belongs to the chemical class of diarylquinolines and was found through whole cell screens of small molecule libraries in *Mycobacterium smegmatis* (Andries et al., 2005). BDQ is bactericidal, selective for mycobacteria, and efficacious against multi-drug resistant and hypoxic, non-replicating *Mtb* (Andries et al., 2005; Koul et al., 2008). In clinical trials, BDQ has been shown to reduce conversion time and prevent further resistance acquisition of MDR-TB when added to standard combination therapy (Diacon et al., 2009; Diacon et al., 2012). It is now a component of multiple phase 2 and 3 clinical trials, and is the most represented drug in novel therapeutic regimens being studied to treat TB (WHO, 2019).

BDQ activity is proposed to work through binding to the c and ε subunits on the ATP synthase, which inhibits ATP synthesis that is essential for *Mtb* survival (Tran and Cook, 2005; Koul et al., 2007; Koul et al., 2008; Biuković et al., 2013; Preiss et al., 2015; Kundu et al., 2016; Sarathy et al., 2019; Saw et al., 2019). Later evidence suggested that at higher concentrations BDQ may also be capable of acting as a protonophore and/or ionophore leading to uncoupling of the ETC *via* collapse of the proton gradient (ΔpH) and membrane potential (ΔΨ), which degrades the PMF and prevents ATP synthesis (**Table 1**) (Feng et al., 2015; Hards et al., 2015; Hards et al., 2018). Inhibition by BDQ depletes intracellular ATP levels (Koul et al., 2007) and induces a significant increase in respiration, likely through activation of inefficient, non-proton pumping components (Cytochrome *bd* oxidase (Cyt-*bd*) and type II NADH dehydrogenase (NDH-2)) of the ETC (Lamprecht et al., 2016). BDQ challenge also upregulates expression of genes encoding for Cyt-*bd* and NDH-2, as well as a set of genes known to be part of the oxidative stress response (Koul et al., 2014; Hards et al., 2015; Lamprecht et al., 2016). However, it is still unclear to what degree direct inhibition of ATP synthesis, loss of the PMF, or increased ROS from enhanced respiration leads to cell death. As well, which of these mechanisms of action is predominant may depend on the concentration of BDQ (Hards et al., 2018).

The continued success of BDQ in clinical trials and its accelerated FDA approval are important milestones in the development of novel anti-TB drugs, however, BDQ is not without its drawbacks. It has been found to have delayed bactericidal activity (3–5 d) *in vitro* (Koul et al., 2014) and *in vivo* (Rustomjee et al., 2008), which appears to be mediated by the metabolic remodeling that upregulates NDH-2 and Cyt-*bd* (Koul et al., 2014). Clinically, its prescription comes with a black box warning for arrhythmias caused by QT prolongation (Fox and Menzies, 2013), and resistance to BDQ was also observed within three years of implementation due to the lack of companion drugs for effective combination therapy (Veziris et al., 2017). In recent years efforts were intensified to improve some of BDQs properties (lipophilicity, long terminal half-life, inhibition of cardiac potassium channel protein (hERG)) by medicinal chemistry (Sutherland et al., 2019). This has yielded compounds with reduced lipophilicity and attenuated hERG blockade, yet maintaining good anti-tubercular activity (Sutherland et al., 2019). A number of recent studies have also identified new inhibitors with novel mechanisms of action at the ATP-synthase (Tantry et al., 2017; Kumar et al., 2018; Saw et al., 2019; Hotra et al., 2020). Clinical trials will have to show if these compounds can replace BDQ in TB chemotherapy in the future. Recent *in vitro* evidence also suggests that BDQ may interfere with the efficacy of first line chemotherapeutics (Shetty and Dick, 2018; Lee et al., 2019; Zeng et al., 2019), as discussed later in this review.

### Cytochrome *bcc:aa_3_* Oxidase

The Cyt-*bcc*::*aa*_3_ complex (encoded by *qcrCAB-ctaBCDE*) is the primary aerobic terminal oxidase in *Mtb* (Cook et al., 2014). It was initially presumed essential *via* early transposon mutagenesis screening for genes essential during growth (Sassetti et al., 2003) and the inability to obtain a deletion mutant (Matsoso et al., 2005). However, recent evidence demonstrates that Cyt-*bcc*::*aa_3_* can be deleted specifically under conditions where glycerol is present, however, knockout strains have a severe growth defect (DeJesus et al., 2017; Beites et al., 2019). Despite this, Cyt-*bcc*::*aa_3_* appears to be quite promiscuous in anti-mycobacterial whole cell drug screens and has led to the identification of several small molecule inhibitors that are efficacious against *Mtb* (Maddry et al., 2009; Moraski et al., 2011; Mak et al., 2012; Pethe et al., 2013; Arora et al., 2014; Rybniker et al., 2015; Chandrasekera et al., 2017; Chong et al., 2020).

In particular, derivatives of an imidazopyridine (IP) scaffold has been shown to be particularly potent against *Mtb*. Early studies looked at certain IP derivatives as anti-mycobacterials, but the compounds were either ineffectual or cytotoxic (Anaflous et al., 2004; Kasimogullari and Cesur, 2004; Maddry et al., 2009). In 2011, Moraski et al. identified a carboxyamide derivative that was found to have low micromolar inhibition against *in vitro* replicating, non-replicating, and MDR *Mtb*, without observable mammalian cytotoxicity (Moraski et al., 2011). Mechanistic investigation by transcriptional profiling demonstrated downregulation of energy generation and significant upregulation of the alternative terminal oxidase Cyt-*bd*, but no target was initially identified (Moraski et al., 2011). Sequencing analysis of similar IP compounds later showed QcrB as the most likely target (Abrahams et al., 2012). Pethe et al. then identified an imidazopyridine amide derivative (Q203, Telacebec) that is the current best-in-class compound and is in phase 2 of clinical trials (Pethe et al., 2013). Q203 possesses low nanomolar inhibition of MDR and XDR clinical isolates, high selectivity for mycobacteria, low mammalian cell toxicity, and promising pharmacokinetics (Pethe et al., 2013).

Another promising compound, the proton pump inhibitor Lansoprazole (LPZ, Prevacid), was found in a host whole-cell screen of already FDA-approved drugs (Rybniker et al., 2015). LPZ inhibits the H^+^/K^+^ ATPase pump in gastric mucosa and is approved to treat gastroesophageal reflux disease (GERD). LPZ was found to be a prodrug that is converted to LPZ-sulfide (LPZS) in the host cell cytoplasm and protects from *Mtb* infection (Rybniker et al., 2015). This metabolite was identified as an inhibitor of QcrB, but at a site distinct from that targeted by IP compounds (Rybniker et al., 2015). LPZS was also shown to lack cytotoxicity and to significantly reduce bacterial burden of *Mtb* in a mouse model of infection (Rybniker et al., 2015). Clinically, a population-based cohort study in the United Kingdom found that users of lansoprazole, prescribed for GERD, had lower rates of TB disease than those prescribed alternative proton pump inhibitors (Yates et al., 2017).

The QcrB inhibitors are high affinity antagonists which appear to completely disrupt respiratory activity through the Cyt-*bcc:aa_3_* branch (**Table 2**) (Lamprecht et al., 2016; Kalia et al., 2017). Further investigation of Q203 found that, similar to BDQ, it reduces ATP levels and actually dramatically increases the oxygen consumption rate (OCR) (Lamprecht et al., 2016; Kalia et al., 2017). However, inhibition of the Cyt-*bcc::aa_3_* in a Cyt-*bd* mutant completely abrogates respiration (Arora et al., 2014; Lamprecht et al., 2016; Kalia et al., 2017). Lamprecht and coworkers proposed that Q203-induced reduction in ATP levels causes increased central carbon metabolism and NADH production, activation of Cyt-*bd*, and compensatory respiration in order to maintain oxidative phosphorylation (Lamprecht et al., 2016). Q203 and other imidazopyridine scaffolds have also been found to lead to mild acidification of the internal pH, but interestingly this does not appear to be due to uncoupling activity (Lamprecht et al., 2016; O’Malley et al., 2018). It was subsequently shown that Cyt-*bcc::aa_3_* and Cyt-*bd* share a synthetic lethal relationship: single inhibition of one of the cytochrome oxidases is non-lethal, however, dual inhibition leads to cell death, *in vitro* (Kalia et al., 2017). Furthermore, this combined therapeutic approach was proven significantly more effective than monotherapy with either BDQ or Q203 during *in vivo* macrophage and mouse models of infection (Kalia et al., 2017; Beites et al., 2019).

**Table 2:** Table of bioenergetic inhibitors and their known biochemical disruptions.

Drug	Targets	Static/Cidal	Hypoxia	Starvation	ATP Levels	PMF	Respiration	CCM (ECAR)	NADH/NAD	ROS
Clofazimine	NDH-2	Cidal	Attenuated	**ND**	Decreased/No Change	Yes	Inhibited	Decrease	No change	Yes
Phenothiazines	NDH-2	Cidal	Effective	**ND**	Decreased	Yes	Inhibited	**ND**	Increased	**ND**
Q203	Cyt-*bcc*::aa3	Static	None	None	Moderately Decreased	No	Increased	Increase	No change	No*
Dual Oxidase Inhibition	Cyt-*bcc*::aa3 / Cyt-*bd*	Cidal	Effective	Effective	Decreased	**ND**	Inhibited	**ND**	**ND**	**ND**
Bedaquiline	ATP Synthase	Cidal	Enhanced	None	Decreased	Yes	Increased	Increase	Increased	No*
Menaquinone Inhibitors	Menaquninone Biosynthesis	Cidal	**ND**	Enhanced	Decreased	ND	Inhibited	**ND**	**ND**	**ND**
SQ109	Membrane/Menaquinone	Cidal	Effective	Effective	Decreased	Yes	Inhibited	**ND**	**ND**	**ND**
Nitroimidazoles	Membrane/Cell wall/NO	Cidal	Effective	**ND**	Decreased	ND	Increased	**ND**	**ND**	Yes
Nitazoxanide	Membrane /NO	Cidal	**ND**	**ND**	**ND**	Yes	**ND**	**ND**	**ND**	Yes
Pyrazinamide	Membrane/CoA Synthesis	Cidal	Enhanced	Enhanced	Decreased	Yes	Inhibited	**ND**	**ND**	**ND**

List of bioenergetic inhibitors representative of various drug classes and an accounting of their previously demonstrated effects on bioenergetics, including ATP levels, PMF, oxygen consumption rate (OCR), central carbon metabolism (CCM) as measured by extracellular acidification rate (ECAR), NADH/NAD ratios, and reactive oxygen species generation (ROS). ND, Not Determined; *Timing of ROS measurements may not have captured ROS production. Data collected from: (Amaral et al., 1996; Boshoff et al., 2004; Wade and Zhang, 2004; Weinstein et al., 2005; Huang et al., 2007; Koul et al., 2008; Rao et al., 2008; de Carvalho et al., 2009; Gengenbacher et al., 2010; de Carvalho et al., 2011; Lu et al., 2011a; Lu et al., 2011b; Yano et al., 2011; Li K. et al., 2014; Li W. et al., 2014; Feng et al., 2015; Berube and Parish, 2017; Hards et al., 2018; Vilchèze et al., 2018; Beites et al., 2019; Beites et al., 2019; Lee et al., 2020).

Recently, high-density transposon mutagenesis and genetic deletion of Cyt-*bcc::aa_3_* encoding genes showed that this complex is non-essential, but does result in a severe growth defect (DeJesus et al., 2017; Beites et al., 2019). These observations emphasize the plasticity of the *Mtb* ETC, and its ability to reroute electron flux through the semi-redundant Cyt-*bd* (Arora et al., 2014), leading to bacteriostatic rather than bactericidal inhibition during monotherapy (Lamprecht et al., 2016; Kalia et al., 2017). Even though the recent advances indicate that both terminal oxidases need to be inhibited in concert for a potent bactericidal effect (Kalia et al., 2017; Beites et al., 2019), preliminary clinical data indicates that Q203 has potential as a companion drug. In a recent clinical phase 2 study, increasing doses of Telacebec over 2 weeks in combination with standard therapy were associated with greater reductions in viable mycobacterial sputum load and daily increases in log_10_ time to positivity (deJager et al., 2020). This justifies a clinical phase 3 trial and opens the door to a potentially first all-new pan-tuberculosis regimen of the 21st century (deJager et al., 2020).

### Cytochrome *bd* Oxidase

Growing evidence suggests that the limitations of BDQ and Q203 are due in part to the inherent plasticity of the ETC in *Mtb* (Arora et al., 2014; Koul et al., 2014; Lamprecht et al., 2016; Berube and Parish, 2017). A major component of that flexibility is the alternative terminal oxidase Cyt-*bd* (*cydAB*). Current knowledge about bacterial Cyt-*bd* is primarily based on evidence gained from studies in *Escherichia coli*. Cyt-*bd* in *E. coli* has: (1) a high peroxidase activity (Borisov et al., 2013; Al-Attar et al., 2016), (2) a quick dissociation rate with nitric oxide (NO) (Mason et al., 2009), (3) a low efficiency, and (4) a high affinity for oxygen (Hill et al., 1994), relative to the primary Cytochrome *bo_3_* oxidase. In *Mtb*, Cyt-*bd* is localized in an operon with CydDC, a reductant transporter, that is required for functional assembly of Cyt-*bd* (Pittman et al., 2002; Pittman et al., 2005; Holyoake et al., 2016). In *M. smegmatis*, Cyt-*bd* was found to be important for growth under hypoxic conditions (Kana et al., 2001) and capable of partially compensating for the loss of Cyt-*bcc::aa_3_* (Matsoso et al., 2005). In *Mtb*, transcription of the *cydABDC* operon has been found to be upregulated under oxidative stress (Voskuil et al., 2011), nitrosative stress (Shi et al., 2005; Voskuil et al., 2011), and hypoxia (Voskuil et al., 2004; Shi et al., 2005). As noted above, Cyt-*bd* may also play an important role in *Mtb*’s natural drug tolerance, particularly drugs that directly target the ETC (Matsoso et al., 2005; Small et al., 2013; Koul et al., 2014; Mascolo and Bald, 2019). Indeed, Berney et al. found that inactivation of Cyt-*bd* in *Mtb* abolished the delay in bactericidal activity of BDQ (Berney et al., 2014), and Kalia et al. demonstrated that genetic deletion of *cydAB* caused Q203 to become bactericidal (Kalia et al., 2017). It has also been shown that genetic inactivation of both Cyt-*bcc*::*aa*_3_ and Cyt-*bd* was lethal (Beites et al., 2019). Importantly, this synergy is not restricted to bioenergetic inhibitors as recently a Cyt-*bd* mutant in *Mycobacterium bovis* BCG was found to be hypersusceptible to INH therapy (Zeng et al., 2019). Moreover, inactivation of Cyt-*bd*, by transposon insertion in *cydDC*, required for Cyt-*bd* biogenesis (Moosa et al., 2017), was found to impair both antibiotic tolerance to INH (Dhar and McKinney, 2010) and maintenance of chronic infection (Shi et al., 2005) in murine models. These observations underscore the dramatic role that this previously overlooked, non-essential enzyme may play in protecting *Mtb* from therapeutic challenge and thus its value as a drug target in combination therapy to reduce treatment timelines in TB chemotherapy.

Until recently there were only two Cyt-*bd* inhibitors that had been identified: Aurachin D and Microcin J25 (MccJ25). However, now a third small molecule, ND-011992, has been discovered, promising progress in development of drugs which might enhance the efficacy of other bioenergetic inhibitors (Lee et al., 2020). Aurachins are a family of natural products produced by *Stigmatella aurantiaca* that were found to inhibit both mammalian mitochrondria (Kunze et al., 1987) and bacteria. Further investigation found that while other aurachins non-selectively inhibited all complex III cytochrome oxidases in *E. coli*, aurachin D specifically inhibited Cyt-*bd* (Meunier et al., 1995). Translation of this work in *M. smegmatis*, by Lu et al., found that aurachin D lead to a dose-dependent inhibition of oxygen consumption up to 50% in membrane vesicles, but had no effect on the growth of mycobacterial whole cells (Lu et al., 2015). A follow up study then found that when combined with Q203, Aurachin D abolished respiration in membrane vesicles and potentiated killing in whole cell *Mtb* (Lu et al., 2018). However, further development of Aurichin D is complicated by its toxicity, lack of selectivity, and lipophilic nature (Meunier et al., 1995; Dejon and Speicher, 2013; Li et al., 2013; Schäberle et al., 2014; Lu et al., 2018).

Microcins are post-translationally modified anti-microbial peptides produced by gram-negative organisms (Duquesne et al., 2007). MccJ25 is a low molecular weight peptide naturally produced by the AY25 *E. coli* strain. It is active against foodborne pathogenic bacteria, such as *Shigella* spp., *E. coli* O157:H7 and *Salmonella* spp. (Salomón and Farías, 1992). This biomolecule was first discovered to interact with *Mtb* as a novel inhibitor of the RNA polymerase *in vitro* (China et al., 2011). More recently, MccJ25 was found to selectively inhibit growth and respiration in an *E. coli* strain that only expressed Cyt-*bd*-I (ΔCyt-*bo_3_*/ΔCyt-*bd*-II) (Galván et al., 2018), and also to inhibit purified Cyt-*bd*-I from *E. coli* (Galván et al., 2019). Inhibition by MccJ25 was also found to increase ROS production, which may give a clue as to its mechanism of action (Galván et al., 2018; Galván et al., 2019). Its effects on the mycobacterial Cyt-*bd* is unclear, but worthy of further investigation.

Finally, Pethe and colleagues recently reported discovery of a small-molecule, ND-011992, that was identified in a whole-cell screen based on the conditional essentiality of Cyt-*bd* during Cyt-*bcc*::*aa_3_* inhibition with Q203 (Lee et al., 2020). They demonstrated through biochemical and transcriptomic experiments that ND-011992 inhibits Cyt-*bd* and in combination with Q203 is capable of killing replicating and NRP mycobacteria, as well as enhancing Q203 efficacy in a mouse model of infection (Lee et al., 2020).

These observations suggest a previously unappreciated role of Cyt-*bd* in protection of the cell from hypoxic, oxidative or chemotherapeutic stress. However, it is important to note that its role in protection from hypoxia and oxidative stress has yet to be directly demonstrated in *Mtb*. Furthermore, while its role in protection from drug challenge is clearer, there remains a paucity of lead compounds identified thus far which might progress to clinical trials. And so, study of this unique enzyme remains a promising area for further research to help develop novel strategies to augment TB chemotherapy.

### NADH Dehydrogenases

Another set of enzymes proposed to be important in respiratory flexibility and the target of intense drug discovery efforts is the family of NADH:menaquinone oxidoreductases, also known as NADH dehydrogenases. These enzymes are responsible for oxidative recycling of the TCA cycle product NADH back to NAD+, which is coupled to the reduction of menaquinone to menaquinol (Cook et al., 2009). In *Mtb* there are three NADH dehydrogenases which have been identified (Weinstein et al., 2005). There is one Type-1, proton-pumping NADH dehydrogenase (NDH-1, encoded by the *nuo* operon), and two Type-2, non-proton pumping NADH dehydrogenases (NDH-2): Ndh (encoded by *ndh*) and NdhA (encoded by *ndhA*) (Weinstein et al., 2005; Cook et al., 2009).

NDH-1 has been found to be non-essential under normal and hypoxic, non-replicating conditions (Rao et al., 2008; Vilchèze et al., 2018). However, deletion of *nuoG* was found to prevent *Mtb*-mediated inhibition of macrophage apoptosis (Velmurugan et al., 2007). Similarly, for NdhA, transposon mutagenesis screens demonstrated that it is dispensable for growth both *in vitro* and in an *in vivo* infection model of severe combined immunodeficiency (SCID) mice (McAdam et al., 2002; Sassetti et al., 2003; DeJesus et al., 2017). In the case of Ndh, as with Cyt-*bcc*::*aa_3_*, it was initially presumed to be essential for growth due to the inability of multiple groups to obtain a deletion mutant in *Mtb* (Weinstein et al., 2005; Awasthy et al., 2014; Vilchèze et al., 2018). However, Δ*ndh* mutants were eventually obtained in *M. smegmatis* (Miesel et al., 1998), BCG (Vilcheze et al., 2005), and *Mtb* with significant growth defects (DeJesus et al., 2017).

Multiple studies have probed the different roles that each NDH may play *via* a combination of deletion mutants and chemical genetics. Early on, the antipsychotic drug class of phenothiazines (PTZ) were found to inhibit *Mtb* and arrest respiration (**Table 2**) (Amaral et al., 1996). It was observed that PTZ inhibition of *Mycobacterium leprae*, which lacks both NDH-1 and NdhA, was capable of depleting ATP by nearly 99% by inhibiting Ndh (Katoch et al., 1998). It was later shown that PTZs are NDH-2 inhibitors, capable of disrupting both Ndh and NdhA function (Weinstein et al., 2005). Rao et al. then also utilized PTZs and rotenone, an NDH-1 specific inhibitor, to show that only the NDH-2 enzymes are required for maintenance of ATP homeostasis and survival during hypoxic NRP (Rao et al., 2008). Indeed, inhibition of NDH-1 by either chemical inhibition or genetic deletion failed to cause any measurable decrease in ATP or survival defect in hypoxic conditions (Rao et al., 2008). Meanwhile, chemical inhibition of NDH-2 increased the NADH/NAD+ ratio 2.65-fold, collapsed the ΔΨ, and decreased ATP levels by nearly two orders of magnitude (Rao et al., 2008). Vilcheze et al. then studied an array of NDH single and double mutants to more closely dissect the physiologic roles of each enzyme, but were unable to obtain either an NDH-2 double mutant or an NDH triple mutant (Vilchèze et al., 2018). They found that all Δ*ndh* mutant variants were hypersusceptible to oxidative stress and displayed attenuated virulence *in vivo*, with the Δ*ndh*Δ*nuo* double mutant being the most attenuated (Vilchèze et al., 2018). Most recently, Beites et al. was able to generate a Δ*ndh*Δ*ndhA* double mutant by growing transformants in minimal media without long chain fatty acids (LCFA) (Beites et al., 2019). They confirmed that an NDH-2-deficient strain has elevated NADH/NAD+ ratios, and that rotenone inhibition of NDH-1 in this mutant strain was lethal. Interestingly, they found that when treated with valinomycin across a narrow concentration window, growth on LCFA was partially rescued, but the NADH/NAD+ ratio was not significantly reduced (Beites et al., 2019). The authors suggest that inhibition of the Δ*ndh*Δ*ndhA* mutant on LCFA under aerobic conditions may be due to some form of membrane hyperpolarization caused by overutilization of the proton pumping NDH-1 during aerobic growth on highly reduced carbon substrates that is relieved by valinomycin (Beites et al., 2019). Beites et al. also confirmed prior findings that dual inactivation of *ndh* and *ndhA* lead to death under hypoxic conditions, as well as significant attenuation during *in vivo* infection (Beites et al., 2019). This collection of data indicates that Ndh is likely the primary NADH dehydrogenase in *Mtb* and is essential for maintenance of NADH recycling and ATP homeostasis during hypoxic, non-replicating persistence and during growth on reduced substrates.

There are two major drug classes identified that have been proposed to interact with NADH dehydrogenases in *Mtb*: PTZ and riminophenazines (RPZ), the latter of which includes Clofazimine (CFZ). Importantly, both of these have been shown to be effective in the treatment of MDR-TB (Reddy et al., 1996; Amaral and Viveiros, 2017). PTZs are part of a drug-class called typical antipsychotics, and their effectiveness against TB infection was first reported in clinical case reports that observed disease burden improvement in patients with concurrent psychosis who received phenothiazines (Fisher and Teller, 1959). Two PTZ derivatives, chlorpromazine and thioridazine (TZ), were found to be effective against drug susceptible and MDR-TB in the low micromolar range *in vitro* (Crowle et al., 1992; Amaral et al., 1996). PTZs completely inhibited activity of recombinantly expressed and purified Ndh and NdhA (Weinstein et al., 2005; Yano et al., 2006). These compounds were further shown to be synergistic with INH and RIF and prevent resistance acquisition (Crowle et al., 1992; Viveiros and Amaral, 2001). While the *in vitro* concentrations required to achieve these effects were supratherapeutic (Bettencourt et al., 2000), it has been established that the concentration required to kill intracellular *Mtb* is much lower and may be due to the drugs being concentrated inside the macrophage (Crowle et al., 1992; Ordway et al., 2003). Although, recent work has called into question whether PTZ’s clinical effectiveness is due to NADH inhibition alone or a broader range of effects that disrupt cell wall biosynthesis (Dutta et al., 2011), inhibit bacterial efflux pumps (Viveiros et al., 2005), or direct potentiation of macrophages to facilitate clearance (Machado et al., 2016; Amaral and Viveiros, 2017). The most promising of these drugs is TZ, which has a low side-effect profile, and has been found effective in mono- and combination therapy in mice (Martins et al., 2007b; Dutta et al., 2014a; Dutta et al., 2014b), as well as in limited clinical trials to treat XDR-TB (Abbate et al., 2011; Udwadia et al., 2011). Improving upon the current state of art, a number of studies have generated novel PTZ derivatives which have lower MICs and reduced neurogenic activity (Martins et al., 2007a; Salie et al., 2014; He et al., 2015). However, important work remains to be done to bring PTZ compounds to the TB patient and elucidate their exact mechanism of action.

The second major drug class to interact with NDH is RPZs, the most important of which is CFZ. While these are not actually inhibitors of NDH, they are proposed to require activation by NDH in order to exert their activity. RPZs were first discovered in the 1950s and they were found to delay death in *Mtb* infection models of guinea pigs, enhance respiration in bacteria treated with cyanide (a cytochrome oxidase poison), and were significantly more effective in catalase-deficient *Mtb* (Barry et al., 1957). CFZ was then found to be reduced by purified Ndh in membrane vesicles by acting as an electron acceptor for NADH oxidation and then generating ROS during non-enzymatic re-oxidation by oxygen (Weinstein et al., 2005). Evidence suggests that this activity can also occur, at least to some degree, through NDH-1, but this has yet to be directly investigated (Vilchèze et al., 2018; Beites et al., 2019). CFZ is capable of killing hypoxic NRP cells, decreases central carbon metabolism, inhibits respiration, collapses the PMF, and generates ROS intracellularly (**Table 2**) (Lu et al., 2011b; Yano et al., 2011; Feng et al., 2015; Lamprecht et al., 2016). Interestingly, CFZ has been shown to compete with menaquinone for binding at NDH, circumventing respiration by shuttling electrons from NADH directly to oxygen, and its activity was found to be inhibited by addition of exogenous menaquinone (Lechartier and Cole, 2015). Accordingly, Berube et al. found clofazimine to be particularly effective in combination with a novel menaquinone biosynthesis inhibitor, which may potentiate CFZ reduction at NDH and thus ROS production (Berube and Parish, 2017). Furthermore, CFZ has been shown to shorten treatment duration of first line antibiotics for DS-TB and potentiate second-line drug regimens for DR-TB in *in vivo* murine models of infection (Grosset et al., 2013; Swanson et al., 2015; Tyagi et al., 2015).

In the clinic, CFZ has been restricted because of concerns about its side effect profile, namely orange discoloration of the skin among others (Reddy et al., 1999). However, in an extensive decade-long trial to treat MDR-TB, clofazimine was found to be an integral part of the most effective treatment regimen by facilitating a 99% treatment success rate with 9 months of treatment and no recorded adverse reactions (Deun et al., 2010). Despite this, significant efforts are ongoing to develop RPZ derivatives with better pharmacokinetic and side-effect profiles (Lu et al., 2011b; Zhang et al., 2012a; Zhang D. et al., 2014). One particular analog, TBI-166, is 4-fold more potent *in vitro*, causes less skin discoloration, and is equally effective in murine models of infection as CFZ (Lu et al., 2011b; Xu et al., 2019). TBI-166 is already in phase I clinical trials in China and has also been found to be more effective than first line chemotherapy when used in combination with other ETC inhibitors *in vitro* and *in vivo* (Xu et al., 2019).

There are also a broad host of novel pre-clinical compounds aimed at inhibiting NDH in *Mtb* (Bald et al., 2017; Cook et al., 2017; Foo et al., 2018; Hards and Cook, 2018). Generally speaking, these compounds have been found to have low micromolar to nanomolar affinity, low eukaryotic cytotoxicity, and inhibit oxidative phosphorylation leading to cell death under replicating and non-replicating conditions (Shirude et al., 2012; Heikal et al., 2016; Hong et al., 2017; Harbut et al., 2018; Korkegian et al., 2018; Murugesan et al., 2018; Santoso et al., 2019). Continued development of actual NDH inhibitors may be integral to finding inhibitors that can synergize with current bioenergetic antagonists. However, these compounds may actually antagonize CFZ efficacy as they could inhibit the NADH recycling that generates ROS.

The body of work on NDH in *Mtb*, strongly supports further efforts to characterize the physiologic roles of these enzymes, elucidate the true MOA of existing NDH antagonists, and develop novel NDH inhibitors. While none of the three NDHs are essential, an inhibitor of both NDH-2s or all three NDHs has clear potential to enhance therapeutic efficacy as part of a drug combination. Along those lines, it will be important to understand the exact mechanism of killing of NDH antagonism under various conditions, as ETC disruptions caused by inhibition appears to be significantly different depending on whether the bacteria are under aerobic vs anaerobic conditions (Rao et al., 2008; Beites et al., 2019) and depending on the predominant carbon source (Beites et al., 2019). Drug combinations including NDH inhibitors also need to be explored more closely to ensure avoidance of antagonism that might occur, such as may exist between CFZ and NDH inhibitors that might prevent CFZ reduction.

### Succinate Dehydrogenases

Succinate dehydrogenases (SDH) are important components of the *Mtb* central carbon metabolism and the ETC. SDHs couple the oxidation of succinate to fumarate, with the reduction of menaquinone to menaquinol (Maklashina et al., 2013). In *Mtb*, there are two annotated succinate dehydrogenases (*sdh1* and *shd2)* and one fumarate reductase (*frd*) (Cook et al., 2014), which catalyzes the reverse reaction. Succinate dehydrogenases and fumarate reductases are closely related enzymes whose functions cannot be distinguished from primary sequence alone (Lancaster, 2002). These enzymes have also been shown to have bidirectional activity, so it can often be difficult to differentiate their exact role in energy production and metabolic adaptation for persistence (Lancaster, 2002).

Early transposon mutagenesis screens in *Mtb* reported that *sdh1* was essential for aerobic growth on cholesterol (Griffin et al., 2011), while *sdh2* was essential during hypoxia (Baek et al., 2011). However, the most recent TraSH screen under standard growth conditions showed that none of the *sdh/frd* genes were essential or had growth defects (DeJesus et al., 2017). One of the hallmarks of *Mtb*’*s* response to oxygen limitation and transition to hypoxic NRP is an increased accumulation of succinate both intra- and extracellularly (Watanabe et al., 2011; Eoh and Rhee, 2013). It is currently thought that succinate accumulation acts as a “metabolic battery” to maintain *Mtb*’*s* membrane potential under oxygen-limiting conditions. Succinate accumulation occurs *via* the redirecting of metabolites through the reductive TCA cycle, glyoxylate shunt, and fermentation by fumarate reductase (Watanabe et al., 2011; Eoh and Rhee, 2013). This redirection of metabolism compensates for the reduced oxygen availability and subsequent build-up of reducing equivalents. Through the production and secretion of succinate, unrespired reducing equivalents can be recycled. Under hypoxic conditions *Mtb* was found to increase succinate production 6.5-fold, and secretion was required for maintenance of both the membrane potential and cell survival (Eoh and Rhee, 2013). In a genetic study, Hartman et al. showed that targeted deletion of *sdh1* in *Mtb* prevented the down regulation of respiration in response to decreased oxygen content, inhibited the regrowth of cells after extended stationary phase, and attenuated virulence in mice (Hartman et al., 2014). Deletion of *sdh2* had more modest effects, with a delay in the down regulation of respiration, partial regrowth after extended stationary phase, and no effect on virulence (Hartman et al., 2014). It is important to note that several attempts to simultaneously delete both *sdh1* and *sdh2, or sdh2* and *frdABC* were unsuccessful, suggesting inhibition of all three would further reduce *Mtb* virulence *in vivo* (Hartman et al., 2014).

Despite the central role of succinate dehydrogenases in central carbon metabolism and the ETC, there has been a general hesitation to investigate succinate dehydrogenase inhibitors. This is in part due to the presence of two succinate dehydrogenases, a fumarate reductase and a mammalian counterpart (complex II of the ETC), which complicates inhibitor design. Although there are concerns surrounding Sdh inhibitor design, it does not eliminate these enzymes as useful targets. Sdh enzymes in *Mtb* are different from mammalian Sdh based on their hydrophobic domain and heme content (Lancaster, 2002). Thus, by targeting the regions that differ in the active site, a selective inhibitor may be identified with minimal or no off-target inhibition of complex II.

Although there has been minimal investigation into *Mtb* Sdh inhibitors, the Sdh suicide inhibitor 3-nitropropionate (3-NP) appears to be capable of Sdh inhibition. Inactivation of *Mtb*’*s* succinate dehydrogenases with 200 µM 3-NP lead to a loss in *Mtb* viability during adaptation to hypoxia (Eoh and Rhee, 2013). Treatment of *M. smegmatis* with 3-NP under hypoxia dissipated the membrane potential, supporting the conclusion that Sdh activity is essential for adaptation to hypoxia. Interestingly, 3-NP has also been found to be an inhibitor of isocitrate lyase, an important component of the glyoxylate shunt, but in whole-cells it is presumed to preferentially inhibit Sdh at the membrane (Muñoz-Elías and McKinney, 2005; Dhar and McKinney, 2010; Eoh and Rhee, 2014). Furthermore, the ubiquinone mimic, siccanin, has also been shown to be a species-specific inhibitor of succinate dehydrogenases (Mogi et al., 2009). Siccanin is a potent inhibitor of *P. aeruginosa* and murine succinate dehydrogenases (0.9 µM and 9 µM, respectively), but was less effective against *E. coli* (210 µM) and porcine succinate dehydrogenases (860 µM) (Mogi et al., 2009). Although siccanin activity against *Mtb* remains to be tested, these results demonstrate that there is potential to identify a potent inhibitor of *Mtb* SDHs that does not inhibit mammalian ETC complex II. Furthermore, studies of Cyt-*bd* underscore the value of thorough investigation of possible targets to augment existing chemotherapeutics regardless of essentiality under normal growth conditions. Succinate dehydrogenase inhibitors are likely to work synergistically with other inhibitors targeting ETC components important in maintaining the membrane potential and generation of ATP under hypoxia (e.g. Cyt-*bd*, ATP synthase, NDH). This may ultimately reduce the duration of treatment, prevalence of persistent infections, and delay the development of resistance acquisition.

### Menaquinone

The transport of electrons through the ETC is mediated by lipid soluble electron carriers. In contrast to *E. coli*, which utilizes both ubiquinone and menaquinone, *Mtb* predominately uses menaquinone (Collins and Jones, 1981; Anand et al., 2015). Menaquinone is synthesized from the precursor chorismate *via* a 10-step biosynthetic pathway involving ten enzymes (MenA-J) (Kwon and Meganathan, 2009; Upadhyay et al., 2015). Transposon mutagenesis studies thus far have identified six genes as essential in *Mtb* (DeJesus et al., 2017). Due to the central role of menaquinone in the ETC and the essentiality of oxidative phosphorylation, these enzymes have been the focus of drug screens to identify potent small molecule inhibitors.

Despite the identification of ten potential targets, the majority of inhibitor screening has focused on the final steps in the biosynthetic pathway (MenA, MenB, MenE, and MenG). Several groups have identified micromolar inhibitors with bactericidal activity against both actively replicating *Mtb* and non-replicating *Mtb in vitro* (Dhiman et al., 2009; Kurosu and Crick, 2009; Li et al., 2011). Further investigation into the mechanism of action revealed that inhibition of the menaquinone biosynthetic pathway results in ATP depletion and decreased respiration (**Table 2**) (Sukheja et al., 2017). Inhibition of MenG with the biphenyl benzamide inhibitor DG70, resulted in a >50% reduction in intracellular ATP and inhibition of oxygen consumption (Sukheja et al., 2017). The MenA inhibitor, Ro 48-8071, completely inhibited *Mtb* and *M. smegmatis* oxygen consumption (Dhiman et al., 2009). The depletion of ATP and inhibition of oxygen consumption suggests that menaquinone inhibitors may have synergistic effects with a wide range of bioenergetic inhibitors. Although these inhibitors show promising results *in vitro*, their efficacy *in vivo* remains to be tested. Furthermore, the cytotoxicity of these compounds remains largely unknown and requires further investigation. Kurosu et al. identified a MenA inhibitor that was subsequently found to be cytotoxic due to the presence of benzophenone, but Debnath et al. reported modified benzophenone compounds with reduced cytotoxicity *in vitro* (Kurosu and Crick, 2009; Debnath et al., 2012).

One of the most promising novel menaquinone inhibitors is SQ109, which was initially reported as an inhibitor of MmpL3, an important transporter involved in cell wall biosynthesis (Protopopova et al., 2005; Grzegorzewicz et al., 2012; Tahlan et al., 2012). Since its identification, the exact target of this inhibitor was questioned when activity was found against bacteria that lack a proposed MmpL3 (Protopopova et al., 2005; Sacksteder et al., 2012). It has since been reported that SQ109 also acts as an ETC uncoupler — dissipating the PMF— and as an inhibitor of menaquinone biosynthesis by inhibiting MenA and MenG activity (Li W. et al., 2014; Feng et al., 2015). More recent studies, though, have confirmed that SQ109 is capable of binding MmpL3 in the proton-translocating channel, which may explain its effects on the PMF, however, to what degree these various mechanisms contribute to cell death is still unclear (Li et al., 2019; Zhang et al., 2019) SQ109 treatment of *Mtb* infected mice reduced CFU in the lungs and spleen by 2-log after 28 days, which was comparable to EMB treated mice (Jia et al., 2009). When EMB is replaced with SQ109 in a standard four-drug treatment regimen (INH, RIF, PZA and SQ109) a 32-fold improvement in lung bacterial burden is observed after eight weeks in a mouse model (Nikonenko et al., 2007). Together, these results highlight that SQ109 is an effective treatment for *Mtb* and could be included in current therapeutic drug regimens. Phase II clinical trials with SQ109 have been performed to assess the safety, tolerance, and pharmacokinetics of SQ109 (Heinrich et al., 2015). Treatment with SQ109 alone or in combination with rifampicin over 14 days had no observable bactericidal effects, however, it should be noted the SQ109 bactericidal effects in mice was typically observed after 3–4 weeks (Heinrich et al., 2015). However, another phase II clinical trial found there was no observable difference between standard therapy and regimens containing SQ109 (Boeree et al., 2017). Ultimately further exploration of other treatment combinations is still required to determine if SQ109 can be implemented as an effective *Mtb* treatment.

In addition to inhibiting the menaquinone biosynthetic pathway, direct targeting of menaquinone is also being investigated. This approach involves the use of cyclic peptides which bind directly to menaquinone in order to prevent dependent redox reactions. Hamamoto et al. identified a cyclic peptide, lysocin E, which binds specifically to menaquinone and not ubiquinone, so toxicity to mammalian mitochondria is unlikely (Hamamoto et al., 2014). Lysosin E attenuated *S. aureus* (Hamamoto et al., 2014) and *M. smegmatis* (Yagi et al., 2017) virulence in a silkworm model of infection. Hamamoto et al. also report that lysosin E disrupted *S. aureus* membrane potential and induced cell death (Hamamoto et al., 2014). This suggests that targeting menaquinone in *Mtb* may cause cell death *via* multiple mechanisms: the disruption of electron flux through the ETC preventing ATP production and the direct disruption of the PMF.

The targeting of menaquinone and its biosynthetic pathway is a promising lead for novel *Mtb* inhibitors. The role of menaquinone in growth and survival of both replicating and non-replicating *Mtb* suggests these inhibitors will be potent drugs that can eliminate persistent and drug-resistant infections.

### Broad Mechanism Inhibitors

In addition to the development of target-specific inhibitors of ETC components, several broad mechanism inhibitors have been identified that are capable of disrupting bioenergetic processes, such as PMF and cytochrome oxidase function, as well as other non-bioenergetic cell processes, such as central carbon metabolism and cell wall biosynthesis. These inhibitors have been found to have potent bactericidal effects against *Mtb* and to be broadly capable of clearing NRP cells.

Pyrazinamide (PZA) is currently the gold standard broad mechanism inhibitor for *Mtb* and is a major component of the first-line antibiotic cocktail used for *Mtb* treatment. While the other three inhibitors (isoniazid, rifampin, ethambutol) can be exchanged for alternatives without reducing efficacy, PZA is currently indispensable (Zhang and Mitchison, 2003; Zhang Y. et al., 2014). Several clinical studies have shown that PZA reduces the duration of treatment by 3 months (Aquinas, 1982; Geiter et al., 1987; Fox et al., 1999). This reliance on PZA is largely due to the fact that it is active against slow growing and non-replicating *Mtb* (Heifets and Lindholm-Levy, 1992; Fox et al., 1999; Hu et al., 2006; Vocat et al., 2015). Despite the success of PZA in *Mtb* treatment, the mechanism of action has been difficult to characterize and many mechanisms have been proposed, including inhibition of fatty acid and coenzyme A (CoA) biosynthesis, impairment of *trans*-translation, and collapse of the membrane potential. A brief overview of the PZA mechanisms of action that is relevant to bioenergetics is discussed below, but for an in-depth review of the multiple proposed mechanisms see (Lamont et al., 2020).

PZA, and its bioactive metabolite pyrazinoic acid (POA), have been found to decrease the PMF and ATP levels in *Mtb* (Lu et al., 2011a) and to act synergistically with bioenergetic inhibitors (Zhang et al., 1999). These observations, along with an initial finding that PZA disrupts pH homeostasis and membrane potential at very low pH, lead researchers to propose that POA acts as a protonophore and disrupts the membrane potential in *Mtb* (Zhang et al., 1999; Zhang et al., 2003; Singh et al., 2006; Darby et al., 2013; Njire et al., 2015). However, further investigation found that at a more neutral pH, PZA and POA retained inhibitory activity but did not lead to cytoplasmic acidification or loss of membrane potential in contrast to CCCP, a known protonophore (Peterson et al., 2015). This suggests that protonophore activity is unlikely to be a clinically significant component of PZA activity. Instead, mounting evidence suggests the primary mechanism of action may be through the disruption of CoA biosynthesis *via* inhibition at *panD* and subsequent perturbations of CoA-dependent reactions in central carbon metabolism (Dillon et al., 2014; Shi et al., 2014; Gopal et al., 2016; Gopal et al., 2020; Lamont et al., 2020; Sun et al., 2020). Indeed, as discussed in detail below the TCA cycle, which requires CoA for multiple steps is an essential component of energy metabolism in *Mtb* (Wayne and Lin, 1982; Zhang and Mitchison, 2003; Singh et al., 2006; Baek et al., 2011; Nandakumar et al., 2014; Njire et al., 2015). Of particular importance is that under conditions where energy production is low (e.g. hypoxia, nutrient limitation) the bactericidal effects of PZA are increased (Wade and Zhang, 2004; Hu et al., 2006; Huang et al., 2007). Hu et al. (2006) showed that increasing the length of dormancy increased the bactericidal activity of PZA, while Huang et al. (2007) showed that starvation decreased membrane potential and increased PZA activity. Interestingly, the cellular pools of CoA are also decreased under nutrient starvation and hypoxia (Wade and Zhang, 2004; Hu et al., 2006). As well, *Mtb* strains with mutations in NADH dehydrogenase, nitrate reductase, formate reductase, potassium transport, and NAD recycling also increased the susceptibility of *Mtb* to PZA (Zhang et al., 2013). Furthermore, Wade and Zhang also show that treatment of *Mtb* with BDQ or with ETC uncouplers (CCCP or valinomycin) potentiated the effects of PZA against *Mtb* (Wade and Zhang, 2006). Although the exact mechanisms of action of PZA are still not well understood current knowledge argues that PZA disrupts bioenergetics and acts synergistically with other bioenergetic inhibitors.

Another example of broad mechanism inhibitors of *Mtb* are the nitroimidazoles, pretomanid (PA-824) and delamanid (OPC-67683) (Stover et al., 2000; Matsumoto et al., 2006). These inhibitors were found to inhibit mycolic acid biosynthesis, which is a major component of the *Mtb* cell envelope (Abrahams and Besra, 2016). More importantly, pretomanid inhibits both replicating and NRP cells. This observation was surprising, considering that under non-replicating conditions mycolic acid biosynthesis is downregulated (Wayne, 1994; Betts et al., 2002). This suggested that pretomanid had an additional mechanism of action.

Both pretomanid and delamanid activity requires activation by an F420 nitroreductase enzyme, which produces *des*-nitro metabolites (Singh et al., 2008; Purwantini et al., 2018). The conversion of pretomanid to these metabolites leads to the production of NO, which is capable of poisoning cytochrome oxidases and is likely responsible for the observed anerobic killing (Singh et al., 2008; Gurumurthy et al., 2011). Interestingly, the development of *Mtb* resistance to these inhibitors occurs *via* mutations that disrupt F420 biosynthesis preventing drug activation (Singh et al., 2008). Although the acquired mutations increase resistance, strains with disrupted F420 biosynthesis have been shown to be hypersensitive to killing by NO (Gurumurthy et al., 2012). Thus, coupling pretomanid or delamanid with another drug that induces NO production or impairs protective mechanisms, would likely have synergistic effects and would reduce the risk of resistance acquisition. Furthermore, pretomanid treatment causes a rapid drop in intracellular ATP levels, increased menaquinol/menaquinone ratio, and increased expression of cytochrome *bd* oxidase and nitrate reductase (*narGHIJ*) (Manjunatha et al., 2009). It is evident that pretomanid is an exciting new drug for the treatment of *Mtb*, and that further inhibition of bioenergetics in combination may improve treatment.

A recent phase III clinical trial found a 90% treatment success in XDR-TB or MDR-TB infected patients treated with pretomanid, bedaquiline and linezolid (Conradie et al., 2020b). Meanwhile, delamanid is currently approved for the treatment of XDR-TB and MDR-TB patients. Early phase II clinical trials of delamanid reported similar bactericidal activity as rifampicin over two weeks (Diacon et al., 2011). The inclusion of delamanid in a background drug regimen for treatment of MDR-TB also significantly improved culture conversion rates when combined with background drug regimens compared to placebo (Gler et al., 2012; Skripconoka et al., 2012). However, a recent phase III clinical trial of delamanid found no difference in outcomes between the addition of delamanid to a WHO-recommended optimized treatment and the addition of a placebo (vonGroote-Bidlingmaier et al., 2019). This may suggest that efficacy of delamanid, and possibly pretomanid, is dependent on companion drugs used in the regimen. Further investigation of the use of delamanid and pretomanid for XDR-TB and MDR-TB patients is therefore required to determine its potential role in the clinical setting.

Nitazoxanide (NTZ) is another example of a broad mechanism inhibitor and a repurposed FDA-approved drug that has previously been identified as a possible drug for *Mtb* treatment. Originally identified for its treatment of *Giardia* and *Cryptosporidium*, NTZ potently inhibits *Mtb* under both replicating and non-replicating conditions (de Carvalho et al., 2009). Furthermore, the authors were unable to obtain any NTZ-resistant mutants (de Carvalho et al., 2009). When screened against 50 clinical isolates of drug susceptible, drug resistant, and multi-drug resistant strains, NTZ was active against all strains (Shigyo et al., 2013). NTZ has been identified as an inhibitor of pyruvate: ferredoxin/flavodoxin oxidoreductases (PFORs) (Hoffman et al., 2006), however, *Mtb* does not have an annotated PFOR suggesting an alternative mechanism of action. de Carvalho et al. showed that NTZ can disrupt *Mtb* membrane potential and pH homeostasis (de Carvalho et al., 2011). In addition to targeting *Mtb*, NTZ has also been found to inhibit mTORC1 signaling resulting in autophagy and enhancing *Mtb* killing (Lam et al., 2012). Further information regarding the mechanisms of action of NTZ in *Mtb* remains to be investigated. It is also important to note that NTZ is inactivated in mouse models (Shigyo et al., 2013). Furthermore, whole blood assays from human subjects have also shown that NTZ and its bioactive metabolite, tizoxanide, have poor anti-mycobacterial activity due to low bioavailability from inactivation and/or protein binding (Harausz et al., 2016). These studies suggest that NTZ possesses poor pharmacodynamic and -kinetic properties (Shigyo et al., 2013; Harausz et al., 2016). Despite this, and because NTZ is already FDA approved, a phase II clinical trial was conducted to determine its efficacy for treating TB in humans. In a 14-day trial in patients with drug-susceptible pulmonary TB, NTZ displayed no bactericidal effects (Walsh et al., 2020). The authors found that plasma levels of NTZ were below the MIC, and there were no detectable levels in pulmonary sputum (Walsh et al., 2020). There has been some success from efforts to improve bioavailability of NTZ derivatives, but the compounds identified to date have also possessed eukaryotic cytotoxicity (Odingo et al., 2017), tempering enthusiasm for the development of NTZ or its derivatives as viable therapeutics to treat TB. (Walsh et al., 2020)

### Combination Therapy With Bioenergetic Inhibitors

Combination therapy is now the standard in all active tuberculosis treatment regimens (WHO, 2017). Unfortunately, most of these combinations are an amalgam of monotherapies whose combinatorial effects have not been closely examined prior to inclusion in clinical trials. Developing more synergistic combination therapies has the potential to lower drug dosages required for treatment, which can reduce both deleterious side effects and incidence of antibiotic resistance in TB treatment regimens (Zimmermann et al., 2007; Lehár et al., 2009). As more and more bioenergetic inhibitors are discovered and ultimately included in chemotherapeutic regimens to treat TB, it is critical that we closely evaluate how these new drugs might interact with each other and existing therapeutic regimens prior to inclusion in costly and lengthy clinical trials. Importantly, growing evidence suggests that bioenergetic inhibitors are broadly effective in combination with each other, as well as existing first-line chemotherapeutics.

Early work on the potential of bioenergetic inhibitors was conducted by Nuermberger and colleagues, who found that BDQ and pretomanid improved *Mtb* lung burden (2.5-log-fold reduction) in a mouse model of infection after 1 month of treatment compared to a combination treatment of rifampicin, isoniazid, and pyrazinamide (Tasneen et al., 2011). Addition of clofazimine to the combination led to a greater than 5-log-fold reduction compared to standard therapy, and both new combination regimens sterilized infection after three months (Tasneen et al., 2011). There are now seven phase II and III clinical trials which include a combination of bedaquiline and either pretomanid or delamanid (WHO, 2019).

Mechanistic *in vitro* work on combinations of bioenergetic inhibitors was performed by Lamprecht et al., who found that a combination of BDQ, Q203, and CFZ was significantly more lethal than RIF/INH (Lamprecht et al., 2016). Detailed analysis showed that this triple-combo led to an increase in respiration, NADH/NAD+ ratio, ROS, and a depletion of ATP (Lamprecht et al., 2016). This led them to hypothesize that Q203 and BDQ related depletion of ATP enhances central carbon metabolism, as measured by the extracellular acidification rate, in an attempt to replace ATP by substrate-level phosphorylation which provided added fuel for CFZ-mediated ROS production at NDH (Lamprecht et al., 2016). Kalia et al. subsequently showed that genetic inactivation of Cyt-*bd* combined with Q203 caused a *de novo* lethality, demonstrating a synthetic lethal interaction (Kalia et al., 2017). Another QcrB inhibitor, TB47, was also shown to synergize with both pyrazinamide and RIF in a mouse model of infection (Lu et al., 2019). Importantly though, it has been shown by Berube et al., that additive and synergistic effects of drug combinations varied significantly between different *Mtb* strains (Berube and Parish, 2017). They also showed that the efficacy of many bioenergetic inhibitor combinations was limited under starvation conditions, however, deletion of Cyt-*bd* reestablished their killing activity (Berube and Parish, 2017).

Cyt-*bd* has quietly become one of the most important and understudied enzymes in anti-mycobacterial drug development. Clear evidence indicates its synergistic role with Q203 (Kalia et al., 2017), and its ability to prevent drug tolerance with bioenergetic inhibitors under hypoxic conditions (Berube and Parish, 2017). It is predicted catalase/peroxidase activity (Borisov et al., 2011; Al-Attar et al., 2016) suggests it may be protective against combinations that are dependent on ROS production, such as those including CFZ (Berube and Parish, 2017), and its predicted NO protection (Mason et al., 2009) could help the cell tolerate challenge with nitazoxanide and nitroimidazoles. An effective inhibitor of Cyt-*bd* like ND-011992 (Lee et al., 2020) may prove to be synergistic with a broad number of bioenergetic inhibitors and should be investigated further.

Multiple studies have also shown that menaquinone biosynthesis inhibitors broadly improve lethality in combination with other bioenergetic inhibitors (Sukheja et al., 2017; Berube et al., 2019). DG70 mediated inhibition of MenG was also synergistic with BDQ, INH or PA-824. The most potent combination (DG70/INH) completely sterilized *Mtb* cultures in an *in vitro* persistence model (Sukheja et al., 2017). Berube et al. demonstrated that dual treatment of NM-4 (a MenA inhibitor) with sub-bactericidal concentrations of BDQ, CFZ, or ND-10885 (an imidazopyridine inhibitor of Cyt-*bcc*::*aa_3_*) had synergistic effects *in vitro* and sterilized *Mtb* within 21 days. The most potent combination was ND-10885/NM-4, which caused near complete sterilization within 7 days (Berube et al., 2019). These data further demonstrate a complex network of interactions between enzymes in the ETC, and the exciting prospect that combinatorial inhibition may have for the improvement of anti-TB therapy.

Significant work has also focused on the effects that bioenergetic inhibitors have on first-line drugs. SQ109 was found to be synergistic with INH, RIF, and PZA, showing a 32-fold improvement over the current standard treatment in mice (Nikonenko et al., 2007). However, a growing body of evidence suggests that bioenergetic inhibitors are also capable of significantly antagonizing standard drug regimens. Recently, several *Mtb* cell wall synthesis inhibitors (INH and EMB) have been shown to induce an ATP burst in *M. bovis* BCG that was required for lethality and attenuated by addition of sub-MIC concentrations of BDQ or CCCP (Shetty and Dick, 2018). Another study similarly showed that BDQ prevents INH and moxifloxacin mediated ATP burst, which results in complete inhibition of INH-induced cell death while delaying moxifloxacin-induced cell death (Lee et al., 2019). Despite these observed antagonistic drug interactions, co-administeration of BDQ, Q203 or CCCP with rifampicin was not antagonistic (Lee et al., 2019). Unlike INH and moxifloxacin, rifampicin does not induce an ATP burst highlighting an alternative cell death pathway that is compatible with bioenergetics inhibitors (Lee et al., 2019; Zeng et al., 2019). Through an unclear alternative mechanism, INH was also found to cause a dose-dependent antagonism with RIF-PZA combination therapy (Almeida et al., 2009). Alternatively, inhibition of menaquinone biosynthesis (Sukheja et al., 2017) is synergistic with INH, highlighting the fact not all bioenergetics inhibitors are antagonistic to current *Mtb* antibiotics. Ultimately these results illustrate that while bioenergetics inhibitors are a promising new addition to combination regimens to kill *Mtb*, careful consideration of drug combinations is required to ensure that antagonistic effects do not occur.

## Mechanisms of Antibiotic-Induced Cell Death

**Figure 2:**
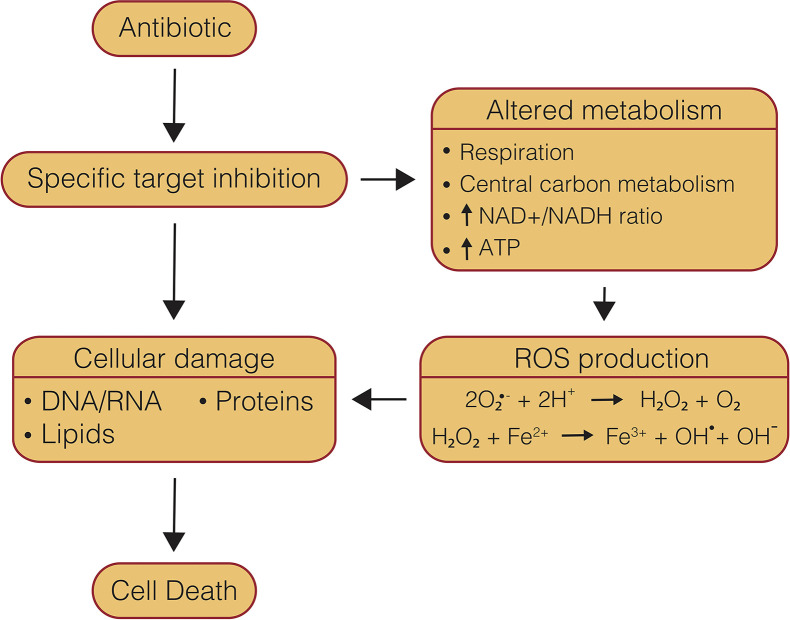
Mechanisms of conventional, bactericidal antibiotic-induced cell death. Conventional, bactericidal drugs have multiple components of their mechanisms of action which lead to cell death, beginning with specific target inhibition, which leads to damaged biomolecules, as well as altered metabolism and ROS production.

Understanding the mechanisms of antibiotics is essential to overcoming obstacles that can present themselves during drug development, such as resistance acquisition, side effects, and antagonism in multi-drug therapy. The mechanism of cell death caused by drugs is often multi-modal, and therefore can be difficult to parse. Several major mechanistic themes have been identified as part of antibiotic-induced killing of bacteria, including direct target inhibition, generation of ROS, and inappropriate metabolic activity (**Figure 2**). Each of these components can in turn be perturbed by external cellular conditions which may protect or potentiate antibiotic lethality. The presence or absence of these various components can also be a key distinguishing feature that determines antibiotic efficacy, such as whether a drug is bactericidal or is capable of clearing NRP cells. While bacteriostatic drugs can be used successfully to treat bacterial infections, bactericidal drugs offer unique advantages and have been shown to be necessary in the treatment of aggressive infections such as meningitis, osteomyelitis, and endocarditis (Finberg et al., 2004; Pankey and Sabath, 2004; Rhee and Gardiner, 2004). As well, the ability to clear persister cell populations is also essential to reducing treatment timelines (Barry et al., 2009; Cohen et al., 2013). One of the prerequisites for new anti-mycobacterial chemotherapeutics, particularly bioenergetic inhibitors, is the ability to kill NRP cells, which are believed to contribute to prolonged treatment timelines required to cure tuberculosis (Wayne, 1994; Gomez and McKinney, 2004; Cohen et al., 2013; Rittershaus et al., 2013). Direct target inhibition tends to be the primary concern when discussing antibiotic mechanisms of action. Traditional chemotherapeutics, namely first and second-line drugs, generally target biomass generating metabolic pathways, such as DNA replication, RNA transcription, protein translation, and cell wall synthesis (Rittershaus et al., 2013).

In mycobacteria, INH and EMB disrupt mycolic acid biosynthesis, aminoglycosides induce protein mistranslation at the ribosome, RIF inhibits RNA synthesis by the RNA polymerase, and fluoroquinolones prevent DNA synthesis by binding to DNA topoisomerases and gyrase (Rittershaus et al., 2013). Similarly, much of the work characterizing bioenergetic inhibitors has focused on the direct results of target inhibition, as discussed previously. However, growing evidence in the broader microbiology field suggests that target inhibition is only one component of a wider array of mechanisms that contribute to cell death. More recent efforts have focused on understanding the role of reactive oxygen species (ROS) and metabolic activity have in antibiotic lethality. Understanding how and if these mechanisms apply to ETC inhibition will be essential to advancing rational design of combination drug therapies which incorporate bioenergetic inhibitors.

### Reactive Oxygen Species’ Uncertain Role in Bioenergetic Inhibition

As reviewed by Imlay (2013), ROS are a normal byproduct of cellular metabolism. This well-accepted model proposes that auto-oxidation of flavin dehydrogenases leads to production of superoxide and H_2_O_2_ (Messner and Imlay, 1999; Seaver and Imlay, 2004). Superoxide is then capable of poisoning Fe-S cluster-containing enzymes that are essential for scavenging ROS, while H_2_O_2_ undergoes the Fenton reaction with unincorporated iron to generate hydroxyl radicals that can damage DNA, lipids, and proteins (**Figure 2**) (Imlay, 2013; Acker and Coenye, 2017). Bacteria possess constitutive and inducible systems to respond to this ROS production; however, elevated oxidative stress is able to quickly overcome these defenses leading to cell death (Kumar et al., 2011).

It had long been observed that some antibiotics induce cellular redox stress response pathways in bacteria (Kono, 1982; Sherman et al., 1996; Unissa et al., 2016). It was only more recently, though, that antibiotic-induced production of ROS was proposed to be part of a concerted mechanism of antibiotic lethality (Dwyer et al., 2007; Kohanski et al., 2007). Kohanski et al. discovered that treatment with the bactericidal antibiotic classes β-lactams, fluoroquinolones, and aminoglycosides in *E. coli* led to a significant increase in ROS, while the bacteriostatic drug chloramphenicol showed no increase in ROS levels (Kohanski et al., 2007). Increased ROS production was associated with an increase in respiration, consumption of NADH (Kohanski et al., 2007; Lobritz et al., 2015), and the ATP/ADP ratio (Akhova and Tkachenko, 2014). Subsequent metabolomic analysis of these antibiotic classes in *E. coli* found they caused an increase in central carbon metabolism and deleterious products of oxidative stress (Belenky et al., 2015). Furthermore, this production of ROS was associated with DNA, protein and lipid damage (Kohanski et al., 2007; Wang and Zhao, 2009; Wang et al., 2010; Dwyer et al., 2012; Kottur and Nair, 2016; Hong et al., 2019). Addition of an iron chelator, hydroxyl radical quencher, or inactivation of Fe-S cluster biosynthesis attenuated ROS levels and killing for all three drugs, suggesting that ROS production likely occurs through metabolic stimulation and overactivation of the endogenous ROS pathway (Kohanski et al., 2007). These findings led the authors to hypothesize a model where, in addition to target-specific inhibition, bactericidal antibiotics are capable of contributing to cell death *via* the generation of ROS, which leads to oxidative damage of essential cellular components (**Figure 2**) (Kohanski et al., 2007; Foti et al., 2012; Dwyer et al., 2014; Belenky et al., 2015). It is important to note that several studies called this model into question and found that bactericidal antibiotics are capable of killing in the absence of ROS (Ezraty et al., 2013; Keren et al., 2013; Liu and Imlay, 2013). However, a large number of studies have continued to find that, under a wide array of conditions and with a variety of bacteria and bactericidal antibiotics, ROS are at least a component of cell death (Davies et al., 2009; Wang and Zhao, 2009; Calhoun and Kwon, 2011; Foti et al., 2012; Grant et al., 2012; Ling et al., 2012; Liu et al., 2012; Akhova and Tkachenko, 2014; Dwyer et al., 2014; Piccaro et al., 2014). While it is clear from this debate that an array of bacterial and environmental factors can influence antibiotic-induced cell death and associated ROS generation, (Fang, 2013; Kram and Finkel, 2013; Acker and Coenye, 2017; Wescott et al., 2017; Baquero and Levin, 2020) these cumulative data suggest that ROS are likely an important component of the mechanism of killing with these canonical bactericidal antibiotics (Acker and Coenye, 2017; Baquero and Levin, 2020).

**Figure 3:**
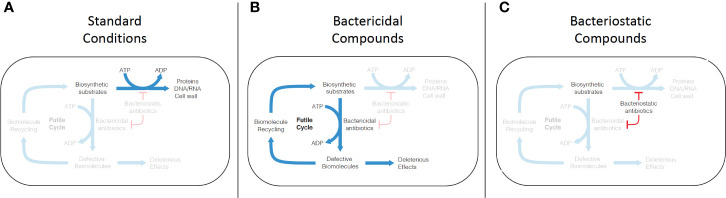
Mechanism of futile cycling induced by defective synthesis from bactericidal antibiotic challenge. **(A)** Under standard growth conditions, biosynthetic substrates (nucleic acids, amino acids, peptidoglycans) are synthesized into biomolecules (DNA/RNA, proteins, cell wall). **(B)** Conventional bactericidal compounds induce defective synthesis (e.g. mistranslation by aminoglycosides), which is proposed to generate an ATP-consuming futile cycle, as well as direct deleterious effects caused by incorporation of some of the defective biomolecules into cellular structures and machinery. **(C)** Bacteriostatic compounds appear to inhibit both standard biosynthesis and defective biosynthesis, disrupting the futile cycle induced by bactericidal compounds.

Of further interest is the finding that ROS generation likely occurs through increased respiration and activation of the endogenous ROS pathway (Kohanski et al., 2007; Dwyer et al., 2014; Yang et al., 2017) This is in line with reports that show one distinguishing factor of bactericidal drugs compared to bacteriostatic drugs is the presence of dominant negative effects that lead to defective synthesis (**Figure 3**), rather than inhibition of synthesis (Davis et al., 1986; Hooper, 2001; Nagel and Chan, 2006; Dwyer et al., 2007; Kohanski et al., 2008; Cho et al., 2014). Indeed, it has been shown that treatment with bacteriostatic inhibitors, which halt synthesis altogether, either pre- or post-treatment with bactericidal inhibitors retarded increases in oxygen consumption and reduced lethality in *E. coli* and *S. Aureus* (Lobritz et al., 2015). A model has hence been proposed that defective, energy-consuming replication, transcription, translation, or cell wall synthesis could lead to a futile cycle, causing a dramatic increase in metabolic demand, central carbon metabolism, and NADH recycling leading to increased respiration and production of ROS (Brynildsen et al., 2013; Cho et al., 2014; Cameron et al., 2018; Stokes et al., 2019) (**Figure 3**). Although, further investigation is required to fully understand this connection between bactericidal antibiotic efficacy and metabolic dysregulation.

Support for this model has also been found in *Mtb*, although to a lesser degree, for bactericidal antibiotics against traditional drug targets. INH and RIF, both bactericidal drugs, have been found to dramatically increase hydroxyl radical formation, while EMB, a bacteriostatic drug, does not (Piccaro et al., 2014; Zeng et al., 2019). It has also been shown that INH causes an increase in respiration (Zeng et al., 2019), while EMB does not (Zeng et al., 2019). INH lethality was also potentiated by inactivation of ROS scavenging enzymes AhpC and SOD (Wilson and Collins, 1996; Dussurget et al., 1998). Grant et al. has further shown that combination therapy with the bactericidal drugs ciprofloxacin and INH lead to the production of persister cells whose population was inversely proportional to levels of dissolved oxygen in culture, strongly suggesting that molecular oxygen is utilized for ROS production which is required for culture sterilization (Grant et al., 2012). ROS production by these antibiotics in *Mtb* has been shown to lead to the production of oxidized nucleotides that contribute to lethality (Fan et al., 2018). Definitive evidence of whether ROS directly contribute to these bactericidal phenotypes or whether they are an associated phenomenon in *Mtb* is still lacking. However, in the context of the work presented above in other organisms, these data suggest that traditional bactericidal chemotherapeutics against *Mtb* stimulate metabolic activity and ROS production, which leads to oxidative stress and likely contributes to antibiotic-induced cell death.

The role of oxidative stress in bioenergetic inhibitor-induced cell death, however, is less clear. Levels of ROS did not increase after 3 days of treatment with either BDQ or Q203 at 300x MIC under aerobic conditions, despite dramatically increasing respiration (**Table 2**) (Lamprecht et al., 2016). While this time point is prior to BDQ bactericidal activity (Koul et al., 2014), and monotherapy with Q203 is bacteriostatic (Kalia et al., 2017), it remains unclear what role, if any, ROS plays in the mechanism of these bioenergetic inhibitors. The primary mechanism of action of CFZ is actually proposed to occur through direct generation of ROS (Lamprecht et al., 2016), but its retained efficacy under anaerobic conditions suggests that this is only one component of its mechanism of action (Lu et al., 2011b). Furthermore, the majority of bioenergetic inhibitors similarly retain efficacy under anaerobic conditions, some of which like BDQ and pyrazinamide are actually improved at low oxygen tensions where ROS generation would be minimal or absent (**Table 2**).

Thus, it becomes clear that the theory of ROS generation as a major mechanism of action of antibiotic-induced lethality may not be universally applied to bioenergetic inhibitors. While this may not be surprising, as they inhibit the respiratory machinery that itself generates ROS, these observations underscore the need to further understand the biochemical implications of bioenergetic inhibitors.

### Metabolic Activity

Another major component affecting the efficacy of conventional bactericidal antibiotics is the pre-existing metabolic state of the cell. It has been frequently observed that metabolically active bacteria are more susceptible to antibiotics (Kohanski et al., 2007; Lewis, 2010; Lee et al., 2018; Stokes et al., 2019), and bactericidal activity is positively correlated with rate of growth (Lee et al., 2018). Conversely, slow growing and metabolically quiescent bacteria are more tolerant to challenge by a wide array of drug classes (Brown et al., 1988; Eng et al., 1991; Spoering and Lewis, 2001; Nguyen et al., 2011). Subpopulations of antibiotic tolerant cells have been found to play a role in treatment-recalcitrant chronic infections (LaFleur et al., 2006; Mulcahy et al., 2010) and are proposed to be responsible for prolonged treatment timelines (McDermott, 1958; Levin and Rozen, 2006). This protection is proposed to occur through reduced abundance of or reliance on the biomass producing enzymes targeted by these antibiotics, and therefore limiting the deleterious effects of defective synthesis and futile cycling (Levin and Rozen, 2006; Lewis, 2010).

Early studies in a variety of gram-negative and gram-positive bacteria indicated that the cell-mediated transition to quiescence and antibiotic tolerance were induced by mechanisms such as the stringent response and TA modules due to stressors like nutrient starvation and hypoxia (Lewis and Manuse, 2019). However, genetic deletions of these systems failed to inhibit antibiotic tolerance, suggesting that while they are capable of inducing quiescence, there are many redundant processes which can compensate for the loss of individual mechanisms (Conlon et al., 2016). Such complex mechanisms make small molecule inhibition of persistence difficult, thus emphasizing the need for drugs which are capable of killing regardless of the pre-existing metabolic state of the bacterium. One reoccurring theme throughout these studies was the observation that quiescence was accompanied by decreased respiration and ATP depletion (Stokes et al., 2019). Along these lines, a number of studies have investigated the effects that disruption of oxidative phosphorylation and the TCA cycle might have on antibiotic tolerance.

Lobritz et al. found that inhibition of bacterial respiration *via* deletion of the cytochrome oxidases in *E. coli* protected against β-lactams, aminoglycosides, and fluoroquinolones (Lobritz et al., 2015). Lewis and Colleagues subsequently discovered that addition of arsenate, which induces a futile cycle of ADP-arsenate synthesis and hydrolysis that competes for PMF utilization and reduces ATP synthesis, increases the persister cell population and antibiotic tolerance in both *S. aureus* (Conlon et al., 2016) and *E. coli* (Shan et al., 2017). Conversely, a nutritionally induced elevation in metabolic states, as measured by ATP levels and regardless of growth rate, led to enhanced bactericidal activity (Conlon et al., 2016; Lopatkin et al., 2019). Interestingly, ATP depletion by either inactivation of the ATP synthase or enhanced hydrolysis *via* overexpression of the F1 subunit of the ATP synthase actually upregulated central carbon metabolism, respiration, and membrane potential in an attempt to increase substrate-level phosphorylation. This resultant increase in metabolic activity enhances antibiotic lethality (Jensen and Michelsen, 1992; Koebmann et al., 2002; Lobritz et al., 2015). The reason why different modalities of ATP depletion can lead to differential susceptibility to antibiotics is unclear. It is possible that bacteria possess graded cellular responses to ATP depletion that lead to either metabolic quiescence or activation depending on the severity of depletion. For example, competitive antagonism of the PMF by arsenate may allow for sufficient ATP synthesis to facilitate a controlled metabolic slow-down, where complete inhibition of ATP synthesis or forced ATP hydrolysis may lead to severe ATP depletion and an emergency response by the cell which upregulates central carbon metabolism in an attempt to compensate *via* substrate-level phosphorylation. Regardless, respiration *via* the ETC and ATP levels appear to be an important modulator of metabolic activity and antibiotic lethality. These observations have led to the development of a model of drug tolerance during low energy states, whereby decreased ATP levels signal downregulation of biomass generating processes thereby reducing levels and/or activity of bactericidal antibiotic targets and minimize their deleterious effects (Zalis et al., 2019).

With regards to the TCA cycle, genetic deletion of the NADH generating steps, which forces carbon flux through the anaplerotic glyoxylate shunt and circumvents oxidative phosphorylation, protected against conventional bactericidal antibiotics (Kohanski et al., 2007). More recent work has confirmed this finding and further shown that disruptions, either stochastic or genetic, of NADH-generating TCA enzyme levels reduces ATP concentrations and membrane potential, significantly enriching persister cell populations (Wang et al., 2018; Zalis et al., 2019). In *E. coli* and *S. aureus*, addition of a variety of glycolytic metabolites, as well as pyruvate, energized the respiratory chain and generated a PMF that enhanced aminoglycoside uptake. Metabolic activation of *P. aeruginosa* by addition of exogenous fumarate also increased NADH production and respiration, leading to enhanced aminoglycoside lethality (Koeva et al., 2017). However, addition of glyoxylate to the mixture overrode this signaling and forced metabolic flux through the glyoxylate shunt, reducing respiration and inhibiting aminoglycoside lethality without reducing intracellular concentrations of the antibiotic (Meylan et al., 2017). These data strongly support a model where active respiration and/or TCA-dependent NADH production is required for lethality with conventional bactericidal antibiotics.

Conventional chemotherapeutics in *Mtb* largely demonstrate the same drawbacks to those in fast-growing organisms. For example, INH, RIF, streptomycin, and ciprofloxacin are all bactericidal in replicating *Mtb*, but are largely incapable of killing nutrient-starved or hypoxic NRP cells (Wayne and Hayes, 1996; Betts et al., 2002; Rao et al., 2008; Gengenbacher et al., 2010; Keren et al., 2011). Current practice is to evaluate the efficacy of novel compounds in this respect by challenging *Mtb* under nutrient-starved or hypoxic conditions (Wayne and Hayes, 1996; Wayne and Sohaskey, 2001; Betts et al., 2002; Boshoff and Barry, 2005), in which even bactericidal drugs against traditional targets are ineffective (Wayne and Hayes, 1996; Betts et al., 2002). Although it is important to note that the Wayne model, often used to generate hypoxic NRP cells, is not complete anaerobiosis and is a fundamentally different form of persistence compared to nutrient starvation. For instance, INH becomes completely ineffective under both nutrient-starvation and hypoxia, but RIF, Streptomycin and moxifloxacin are only ineffective under nutrient starvation and retain mild efficacy under hypoxia (Gengenbacher et al., 2010). Similar to other organisms, *Mtb* is capable a mediating a metabolic downregulation in response to stressors like hypoxia and nutrient starvation, which reduce ATP levels and lead to protection from conventional bactericidal antibiotics. As in other organisms, there are many different stress response mechanisms in TB (Torrey et al., 2016), including TA modules (Ramage et al., 2009) and the stringent response (Primm et al., 2000; Dutta et al., 2019), which can induce persistence, but again loss of these systems does not universally lead to antibiotic susceptibility (Singh et al., 2010; Frampton et al., 2011).

While the reductions in respiration and ATP levels have been found to closely correlate with antibiotic tolerance during hypoxia and starvation (Wayne and Hayes, 1996; Rao et al., 2008; Gengenbacher et al., 2010), direct evidence for the role of reduced oxidative phosphorylation and ATP levels in protection from conventional bactericidal antibiotics in *Mtb* is still lacking. These studies have of course been complicated by the inability to obtain cytochrome oxidase and ATP synthase knockouts until recently. There is, however, growing circumstantial evidence that indicates oxidative phosphorylation is essential for bactericidal drug efficacy. Using chemical genetics, a number of groups have shown that Q203 and sub-lethal doses of BDQ reduce ATP levels and induce INH and Moxifloxacin tolerance (Shetty and Dick, 2018; Lee et al., 2019; Zeng et al., 2019). RIF activity was also reduced by co-treatment with Q203, however, pre-treatment with BDQ was not protective (Zeng et al., 2019). Accordingly, Vilcheze et al. discovered that addition of vitamin C, which stimulates respiration and the Fenton reaction, increases ROS production and potentiates killing by both INH and RIF (Vilchèze et al., 2013; Vilchèze et al., 2017). These data indicate that conventional anti-mycobacterial drug efficacy is similarly dependent on the underlying metabolic state of the cell.

Evidence also exists for the role of TCA cycle activity in antibiotic tolerance in *Mtb*. Early work by Wayne and Li found that the glyoxylate shunt, responsible for bypassing the NADH generating steps of the TCA cycle, was also essential for transition to dormancy, survival under hypoxic conditions (Wayne and Lin, 1982), and maintenance of a chronic infection (McKinney et al., 2000). Metabolomic analysis discovered that rerouting of carbon flux away from the TCA and towards triacylglycerol synthesis was essential for survival under a variety of external stressors and for drug tolerance (Baek et al., 2011). Nandakumar et al. also found that in response to INH, RIF or streptomycin challenge, *Mtb* was found to activate the glyoxylate shunt, decreasing NADH production (Nandakumar et al., 2014). Deletion of the first enzymatic step in the shunt, isocitrate lyase (Δ*icl*) increased oxidative stress and potentiated killing >100-fold (Nandakumar et al., 2014). Thus, as with other microorganisms, inhibition of either respiration or TCA-dependent NADH production and subsequent ATP reduction play a universal role in protection from conventional bactericidal drugs against *Mtb*.

Excitement around bioenergetic inhibitors is fueled in large part by their apparent ability to kill *Mtb* persister cells in both hypoxic and nutrient-starved models of non-replicating persistence, when there is an apparent decrease in central carbon metabolism and oxidative phosphorylation (**Table 2**). Under hypoxic conditions, BDQ was actually more effective (Koul et al., 2008), an NDH-2 genetic knockout died spontaneously (Beites et al., 2019), and CFZ had a 10-fold higher MIC but remained quite effective (Lu et al., 2011b) when compared to standard growth conditions. During nutrient-starvation induced persistence, dual-inhibition of the cytochrome oxidases also showed improved efficacy (Kalia et al., 2017). SQ109 was found to be only mildly attenuated under both nutrient and oxygen limited conditions (Li W. et al., 2014), and PZA showed enhanced efficacy under both conditions (Wade and Zhang, 2004; Huang et al., 2007). Therefore, it becomes clear that recent models developed to understand antibiotic lethality in the context of pre-existing metabolic activity, cannot be readily applied to inhibitors of bioenergetics in *Mtb*.

Furthermore, the observed reliance of conventional bactericidal activity on the induction of an active metabolic state for efficacy does not appear to apply to bioenergetic inhibitors either. NDH-2 inhibition, CFZ, dual cytochrome oxidase inhibition, and BDQ have varying effects on respiration and central carbon metabolism; but they all dramatically reduce ATP levels and appear to collapse the membrane potential (**Table 2**) (Rao et al., 2008; Lamprecht et al., 2016; Kalia et al., 2017; Vilchèze et al., 2018; Beites et al., 2019; Zeng et al., 2019). Surprisingly, BDQ has been shown to increase central carbon metabolism by measurement of the extracellular acidification rate (Lamprecht et al., 2016), a read-out for NADH production, and transcriptional upregulation (Koul et al., 2014), but actually antagonizes INH and moxifloxacin activity (Lee et al., 2019; Zeng et al., 2019).

A robust model for understanding lethality of bioenergetic inhibitors is essential for rationally designed combination therapies. The evidence discussed here suggests models developed for understanding conventional bactericidal antibiotics regarding ROS production and metabolic activity insufficiently explain the effects of bioenergetic inhibition that lead to cell death. Thus, we are required to look for alternative models.

### Bioenergetic Inhibitors Mediate Killing Through Collapse of the Proton Motive Force and ATP Depletion

While there is still much work that remains to be done to obtain even a simple accounting of the metabolic disturbances caused by bioenergetic inhibition (**Table 2**), present evidence encourages us to consider alternative models for cell death. Across all of the bactericidal ETC inhibitors for which we have data, the common findings are a reduction in ATP levels and collapse of the PMF. Early work in this area identified an intrinsic requirement for NADH recycling, PMF maintenance and ATP homeostasis even under non-replicating conditions (Gengenbacher et al., 2010). In fact, both starvation and hypoxia cause a five-fold reduction in ATP levels (Rao et al., 2008; Gengenbacher et al., 2010). Under these conditions, BDQ and *N,N*-dicyclohexylcarbodiimide (DCCD), a classical ATP synthase inhibitor, reduced ATP levels in a dose dependent fashion, which was positively correlated with cell death once ATP was reduced beyond a specific threshold (Gengenbacher et al., 2010). Although, under nutrient starvation BDQ was incapable of depleting ATP to levels shown to cause cell-death with DCCD and was therefore ineffective (Gengenbacher et al., 2010). It is important to remember that both of these inhibitors are capable of uncoupling and so its lethal effects could be due to ATP depletion, collapse of the PMF, or both.

Growing evidence suggests that uncoupling is a major component of many bioenergetic inhibitors’ mechanism of action, leading to the development of a model of cell death based on PMF dissipation (Feng et al., 2015; Hards and Cook, 2018). In this model the role of ATP depletion has been minimized based on observations that ATP reductions do not temporally correlate with death in BDQ challenge (Koul et al., 2014), and that a decrease of ATP below measurable levels in *M. smegmatis* during stationary phase did not result in cell death (Frampton et al., 2011). However, as indicated above some evidence suggests that ATP depletion may correlate with cell death only after a certain threshold is reached. Kalia et al. showed that deletion of Cyt-*bcc*::*aa_3_* was non-cidal, but also incapable of reducing ATP levels to those consistent with BDQ treatment. Concurrent genetic deletion of Cyt-*bd* reduced ATP levels to half of that found with BDQ, and led to superior killing in both an *in-vitro* starvation model and *in vivo* (Kalia et al., 2017). Importantly, this differential reduction in ATP was exploited to successfully identify a novel small molecule inhibitor of Cyt-*bd* (Lee et al., 2020). Furthermore, while BDQ has delayed bactericidal activity despite an immediate decrease in ATP levels, final collapse of the bacterial culture correlates closely with reduction of ATP to below measurable levels (Koul et al., 2014). This data is far from conclusive, but strongly supports continued consideration of the role ATP depletion may play in cell death induced by bioenergetic inhibition along with PMF collapse. An important step in understanding these mechanisms better will be the development of standardized protocols to assess the biochemical disruptions caused by bioenergetic inhibitors that facilitates direct comparison. Regardless of whether cell death is primarily mediated by PMF dissipation, ATP depletion, or a combination, the downstream effects that actually mediate cell death are still a mystery. It is insufficient to say that bioenergetic inhibitors kill through disruption of the PMF or ATP homeostasis simply because these processes have been found to be essential. If we are to eventually understand how bioenergetic inhibitors kill we must redouble efforts to understand what downstream deleterious effects or mechanisms actually precipitate cell death (Baquero and Levin, 2020).

## Conclusions

The development of novel anti-TB combination regimens that increase efficacy and reduce treatment timelines have the ability to improve patient compliance, including limitation of side-effects, reduction of cost, and practical ability to complete treatment (Dorman and Chaisson, 2007). These advancements would likely significantly improve the global TB burden and reduce drug resistance acquisition. The introduction of BDQ almost a decade ago ushered in a new age of optimism for novel drug development, particularly for bioenergetic inhibitors, that might be able to circumvent immovable social, political and economic factors in order to cure TB.

As discussed here, inhibitors have been discovered that target almost every enzyme in the respiratory chain, and early drugs from these classes in clinical trials have shown promising results. The almost universal ability of bioenergetic inhibitors to kill NRP cells provides promise for the development of novel regimens to shorten treatment timelines and cure MDR-TB infections. This enthusiasm is bolstered by many of these bioenergetic inhibitors’ proven efficacy in combination regimens with both first-line therapeutics and other inhibitors targeting the respiratory chain.

However, much work remains to be done in order to understand the exact biochemical disruptions and mechanisms of cell death caused by bioenergetic inhibitors. This condition limits our ability to develop rationally designed combination therapies prior to clinical trials, which is essential to reducing overall development costs and time to approval for effective novel regimens (Tyers and Wright, 2019). Importantly, well-developed models that characterize the mechanism of action of conventional, bactericidal antibiotics, such as defective synthesis, ROS generation, and inappropriate metabolic activity are not readily applicable to bioenergetic inhibitors.

Developing new models will be essential to obtaining a foundational understanding of bioenergetic inhibitors’ mechanisms of action. However, our ability to do so is severely limited by a lack of complete characterization of these inhibitors (**Table 2**). A standardized toolkit built to characterize bioenergetic inhibitor-induced disturbances of NADH/NAD ratios, PMF, respiration, ATP/ADP/AMP ratios, and ROS under standard growth conditions and hypoxic and nutrient-starved NRP, would go a long way to rectifying this situation. Employment of such a toolkit across the field would facilitate comparison of both old and new bioenergetic inhibitors, as well as combination regimens, in order to better describe bioenergetic inhibitors’ mechanisms of action.

From our current understanding, it is clear that bioenergetic inhibitors consistently mediate collapse of the PMF and ATP depletion, which most likely is essential for subsequent cell death. However, it remains unclear why exactly flux through the electron transport chain, maintenance of a PMF, and ATP homeostasis are absolutely required for cell survival under non-replicating conditions. The models regarding ROS generation and defective synthesis (**Figures 2** and **3**) as mechanisms of antibiotic-induced cell death are convincing because their explanations are in a sense logically complete and intuitively satisfying. A cell clearly cannot survive when its essential machinery is damaged beyond repair. Either through programmed cell-death, simple decay, or the inability for measurable regrowth; the cells “perish”. However, models which utilize the loss of bioenergetic functions to explain death during starvation or hypoxia-induced non-replication lack a clear dominant negative effect which explains why the cell is incapable of surviving dormancy in the absence of these processes. Of course, PMF is required for an array of processes including ATP synthesis and transmembrane solute transport (Schoepp-Cothenet et al., 2013; Cook et al., 2014), but it is unclear if and why either of those processes are subsequently required during metabolic quiescence. Further investigation into the consequences of the bioenergetic-inhibitor induced disruption of PMF and ATP homeostasis will be essential to identifying the precipitating causes of cell death. Pursuing these lines of investigation will help speed the development of novel bioenergetic inhibitors and combination therapies that hold significant promise to reduce treatment timelines, prevent antibiotic resistance acquisition, and cure TB.

## Author Contributions

EH, TW, and MB wrote the article. All authors contributed to the article and approved the submitted version.

## Funding

This work was financially supported by NIH grant R01AI139465 and Potts Memorial Foundation.

## Conflict of Interest

The authors declare that the research was conducted in the absence of any commercial or financial relationships that could be construed as a potential conflict of interest.
